# Molecular Archaeology of *Flaviviridae* Untranslated Regions: Duplicated RNA Structures in the Replication Enhancer of Flaviviruses and Pestiviruses Emerged via Convergent Evolution

**DOI:** 10.1371/journal.pone.0092056

**Published:** 2014-03-19

**Authors:** Dmitri J. Gritsun, Ian M. Jones, Ernest A. Gould, Tamara S. Gritsun

**Affiliations:** 1 School of Biological Sciences, University of Reading, Whiteknights, Reading, United Kingdom; 2 Unité des Virus Emergents, Faculté de Médecine Timone, Marseille, France; Saint Louis University, United States of America

## Abstract

RNA secondary structures in the 3′untranslated regions (3′UTR) of the viruses of the family *Flaviviridae*, previously identified as essential (promoters) or beneficial (enhancers) for replication, have been analysed. Duplicated enhancer elements are revealed as a global feature in the evolution of the 3′UTR of distantly related viruses within the genera *Flavivirus* and *Pestivirus*. For the flaviviruses, duplicated structures occur in the 3′UTR of all four distantly related ecological virus subgroups (tick-borne, mosquito-borne, no known vector and insect-specific flaviviruses (ISFV). RNA structural differences distinguish tick-borne flaviviruses with discrete pathogenetic characteristics. For *Aedes*- and *Culex*-associated ISFV, secondary RNA structures with different conformations display numerous short ssRNA direct repeats, exposed as loops and bulges. Long quadruplicate regions comprise almost the entire 3′UTR of *Culex*-associated ISFV. Extended duplicated sequence and associated RNA structures were also discovered in the 3′UTR of pestiviruses. In both the *Flavivirus* and *Pestivirus* genera, duplicated RNA structures were localized to the enhancer regions of the 3′UTR suggesting an adaptive role predominantly in wild-type viruses. We propose sequence reiteration might act as a scaffold for dimerization of proteins involved in assembly of viral replicase complexes. Numerous nucleotide repeats exposed as loops/bulges might also interfere with host immune responses acting as a molecular sponge to sequester key host proteins or microRNAs.

## Introduction

Viruses in the genera *Flavivirus* and *Pestivirus* (family *Flaviviridae*) share a common genome organization and coding strategy [1]. The enveloped virions of 50–60 nm contain single-stranded positive-sense RNA genomes >10,000 nucleotides in length, packed into the capsid structure. The open reading frame (ORF) encodes a single polyprotein that is co-translationally processed into 3–4 structural and 7–8 non-structural proteins. The 5′- and 3′-untranslated regions (5′UTR and 3′UTR) flank the ORFs and are involved in virus translation and replication.

The genus *Flavivirus* is subdivided into 4 ecological subgroups that reflect their association with different hosts and different pathogenesis [2], [3]. Two groups, mosquito-borne flaviviruses (MBFV) and tick-borne flaviviruses (TBFV), circulate in nature between distantly related species of vertebrates (mammals/birds) and invertebrates (mosquitoes/ticks). Mosquitoes and ticks transmit flaviviruses to humans while taking a bloodmeal, often resulting in severe disease. *West Nile virus* (WNV), *Japanese encephalitis virus* (JEV), *Dengue virus* (DENV) and *Yellow fever virus* (YFV) are all major MBFV pathogens, causing human infections on an epidemic scale in tropical and subtropical countries, in association with their natural invertebrate hosts, *Aedes* or *Culex* spp. mosquitoes [4].

TBFV are also human pathogens; their circulation in the environment is entirely dependent on ticks and they are geographically widely dispersed. In contrast to MBFV, the genomes of pathogenic TBFV show significantly higher homology (over 92% at the amino acid level), yet they display a variety of clinical syndromes in humans, including, sub-clinical infections, fever, bi-phasic fever, encephalitis and/or haemorrhagic fever. The major human pathogenic TBFV are *Tick-borne encephalitis virus* (TBEV), *Louping ill virus* (LIV), *Powassan virus* (POWV), *Omsk haemorrhagic fever virus* (OHFV), *Langat virus* (LGTV), *Kyasanur Forest disease virus* (KFDV) and *Alkhumra haemorrhagic fever virus* (AHFV) [5], [6].

The life cycles of viruses in the other two flavivirus groups are limited to single host species and are not associated with human disease. The no-known vector flaviviruses (NKV) have only been isolated from rodents or bats whereas flaviviruses in the fourth group, yet to be classified, do not infect mammalian cells. They replicate only in mosquitoes [7]–[9] and are currently known as insect-specific flaviviruses (ISFV). ISFVs are currently subdivided into two ecological subgroups, associated with either *Aedes or Culex spp*.

Pestiviruses (PVs) infect and cause disease in farmed and wild ruminants, causing economic losses in the farming industry and also threatening many wildlife species. The genus *Pestivirus* is subdivided into *Border disease virus* (BDV), *Bovine diarrhoea virus* types 1, 2 and 3 (BVDV1–3), *Classical swine fever virus* (CSV), *Hog Cholera Virus* (HoCV), *Giraffe pestivirus* (GRFPV) and *Reindeer pestivirus* (RNDPV) [10].

Classified into two distinct genera within the family *Flaviviridae*, the flaviviruses and pestiviruses demonstrate no significant sequence homology. However, using a unique approach to the construction of robust nucleotide alignments we have previously revealed unexpected homology in the 3′UTRs of the MBFV, TBFV, NKV and ISFV that are genetically only distantly related [11]–[17]. We showed that the 3′UTR of the primordial precursor of the genus *Flavivirus* evolved by numerous duplications of a region of about 200 nucleotides (that we termed LRSs ie long repeated sequences) localized in the ORF region that encodes the C-terminal domain of NS5^pol^, the RNA-dependent RNA polymerase (RdRp). We demonstrated that almost intact LRSs were preserved in the TBFV group but only sequence remnants of the LRSs as short direct repeats (DRs) were detected in the MBFV, NKV and ISFV groups. However, although this was clearly relevant to the evolution of these viruses, the biological, epidemiological and pathogenic significance of sequence duplications remained unclear.

Here, using a unique research approach based on the construction of 3′UTR alignments and calibrated algorithms to produce MFold-generated RNA secondary structures and in association with functional studies, we reveal duplicated sequences and associated RNA structures in the enhancer region of the distantly related flaviviruses and pestiviruses. We also identify distinct RNA folding patterns in the conserved regions of the 3′UTR of TBFV species associated with different pathogenetic outcomes. A complex role for duplicated enhancer sequences and RNA conformations is proposed, one in the dimerization of accessory proteins for the efficient assembly of replicase complexes and the second in sequestering numerous factors of antiviral immunity (proteins and miRNA) that are important determinants of virus survival in the natural habitat.

## Materials and Methods

The alignments for 3′UTRs of TBFV, ISFV and pestiviruses were constructed using ClustalX available in the BioEdit suite of programs [18] and further edited manually as described [11], [13], [15]–[17]. Briefly, the regions of homology between distantly related virus species were used as anchors to align other less related sequences. This approach reflects an evolution of untranslated region via numerous deletions, insertions, reiterations and duplications, due to the copy-choice activity of RdRp [19]. Nucleotide sequence homology was established using the Sequence Identity Matrix option of BioEdit [18]. Viruses used for alignments are specified by appropriate abbreviations and accession numbers as indicated in Figures.

The secondary RNA structures for individual virus 3′UTRs were predicted using MFOLD 3.2 accessed at http://mfold.rna.albany.edu/?q=mfold/RNA-Folding-Form [20], [21]. Two MFold parameters, i.e. “maximal distance between paired bases” (MDBPB) and “percent suboptimality” (%S) were calibrated manually. An MDBPB of 60–100 and a %S up to 50% were empirically established to produce comparable images between viruses of distantly related groups and were therefore used for routine analysis. The use of an MDBPB value outside the 60–100 range predicted different structures even between closely related virus strains (not shown) and the limited sequence database of 3′UTRs for each recognised species of the TBFV precluded the use of statistical measures of significance used previously [22], [23]. RNA conformations observed as stem-loops (SLs) and Y-shaped structures were enumerated and annotated with the features revealed by alignment and by experimental data when available.

## Results and Discussion

### Duplicated RNA structures in the 3′UTR of TBFV

The secondary RNA structure predictions for selected TBFV virus species are shown in Figures S1A-F.

**Figure 1:**
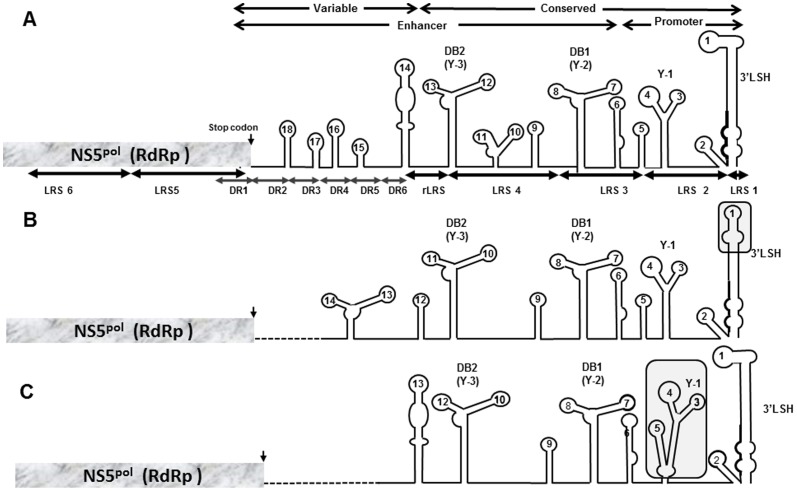
Predicted RNA structures for TBFV. Adapted from the Figure S1 for A) TBEV, B) LGTV and C) OHFV. The 3′UTR and adjacent NS5^pol^ gene region is shown and annotated with the LRSs, DRs, conserved and variable, enhancer and promoter regions. The areas where LGTV and OHFV show different folding are framed.

A comparative alignment of TBFV 3′UTRs (Figure S2) was constructed using TBEV, OHFV, POWV, LGTV, KFDV and LIV. The alignment and corresponding predicted RNA structures (Figure 1, Figures S1 and S2) were annotated with previously identified linear nucleotide motifs that include: 1) LRSs1–6 and short direct repeats (DRs1–6) [16]; 2) variable V3′UTR and conserved C3′UTR regions [24], [25] and 3) boundaries between promoter and enhancer regions [11], [12], [14]. TBFV promoter and enhancer regions have been established empirically as essential and redundant regions for virus replication in tissue culture [15], [26]. The 3′LSH (long stable hairpin) forms part of the tentative flavivirus promoter (Figure 1, Figures S1 and S2) and is highly conserved among TBFV, MBFV and NKV as it interacts directly with NS5^pol^ [27]–[30] and cellular proteins [31], [32]. The conserved pentanucleotide (3′CPN) CACAG at the top of loop 1 has been shown to determine maximal replication efficiency [33]–[35]. Single nucleotide differences in the 3′CPN have been observed between the TBFV/MBFV and NKV groups [36]. The 3′CYCL is another conserved TBFV element which interacts directly with the reverse-complement 5′CYCL sequence localized in the 5′UTR to form a dsRNA panhandle, a second promoter element for the initiation of RNA synthesis [11].

SL2 is a third highly conserved promoter element which plays a key role in disruption of the dsRNA panhandle to facilitate the molecular switch from the circular to linear form of the genome during RNA synthesis [37]. The 3′Y-1 structure, including SL3 and SL4, was lost in the early MBFV precursor lineage that gave rise to the extant MBFV species but has been retained in the NKV and TBFV groups [11], [12], [36]. The hexanucleotide UUGGCA of loop 4 is highly conserved between TBFV and NKV and plausibly interacts with the inverted repeat UGCCAA at the top of 5′SL6, a replication enhancer which maps to the capsid gene [22].The functions of two further promoter elements, Y-1 and SL5, remain to be determined.

The flavivirus enhancer is located between the stop codon and promoter (Figure 1 and Figures S1 and S2). The enhancer is not essential for virus infectivity *in vitro* but its loss attenuates replication, largely due to a reduced level of RNA synthesis [26], [35], [38]–[41]. The enhancer region was proposed to interact with accessory cellular/virus proteins in order to accelerate the assembly of a functional initiator RdRp complex which is likely to be more critical in an environment (than in tissue culture) where the rate of replication determines virus survival in its natural host [14], [22]. The C3′UTR element encodes both promoter and enhancer regions. The V3′UTR encodes only enhancer regions which may differ in length between strains of a virus; spontaneous deletions of long sequence, including the entire V3′UTR, occur during serial laboratory passage of field virus isolates. However, the full-length 3′UTR (about 730 nucleotides) is preserved in wild-type TBFV and is likely to be essential for virus survival in ticks [25], [26], [42].

Although conservation of the TBFV 3′LSH has been reported [24], the 3′LSH in LGTV exhibited altered boundaries for the stem and loop 1 (Figure 1 and Figure S1B). The top of the 3′LSH was distorted due to a single C515T substitution (Figures S1 and S2) which blocked exposure of the 3′CPN - a factor that might contribute to the naturally reduced neurovirulence of LGTV that prompted a human trial of LGTV as a live attenuated vaccine [43]. A similar alteration in the 3′LSH conformation of vaccine (attenuated) strains of YFV 17D (MBFV) has also been observed [44].

Different conformations were apparent in the SL5-SL6 region for OHFV (Figure 1, Figures S1C and S2) and AHFV (available on request). Both of these viruses cause haemorrhagic disease, in contrast to the generally neurovirulent TBFV [5], [6]. It is possible that different sequences and/or folding patterns contribute to the particular pathogenetic outcomes of infection, e.g. via modulation of host innate immune responses [45]–[47] (see also below).

Figure S1 illustrates the homologous structures (Y-2 and Y-4) encoded by TBFV homologous LRS3 and LRS4. The LRS4 and LRS3 were found to be homologous to the short nucleotide regions that encode duplicated dumbbell-like structures DB1 and DB2 [12], [13] observed in MBFV and NKV groups [36], [48]–[50] and because of this evolutionary correspondence, the TBFV Y-2 and Y-4 are referred to here as DB1 and DB2 (Figure 1 and Figures S1 and S2). The A(A/G)AUGGUCG sequence was duplicated in loops 7 and 12 and the GAGA sequence in loops 8 and 13 of the DB1 and DB2 respectively.

The other two species within the TBFV group, LGTV and OHFV, also demonstrated an almost identical pattern of TBEV DB1 and DB2 (Figures S1B and S1C), with similar exposed linear signals, although both of these viruses have shorter 3′UTRs than TBEV. Only one OHFV strain retained DB2; the other two strains probably having lost their DB2s during laboratory passage. The only strain of LIV with an available 3′UTR sequence also seemed to have lost DB2, despite the presence of an intact LRS3, instead forming a long SL structure (Figure S1D). However, two substitutions (C_63_G_64_→G_63_C_64_) restored the LIV DB2 (Figure S1E). The absolute conservation of G_63_C_64_ throughout the TBFV group (Figure S2) suggests that the LIV G_63_C_64_→C_63_G_64_ substitutions might have occurred during laboratory passage of the virus. Alternatively, the absence of the DB2 might be natural and contribute to the milder neuroinvasiveness of LIV in humans in comparison with other TBFVs although direct experimental evidence for this is lacking.

The alignment in Figure S2 shows that POWV (18 strains) and closely related DTV (Deer tick virus, 2 strains) display an identical gap in the V3′UTR, in contrast to the other TBFVs that exhibit gaps of varying length. As it is highly unlikely that an identical RNA region was lost in these 20 viruses, POWV and DTV might demonstrate the naturally shortened length of the enhancer. The entire POWV C3′UTR region was retained, with DB1 and DB2 (Figures S1F and S2), although with loop and bulge sequences different from those of TBEV. The pattern of other repeated sequences also differed between POWV and other TBFV. Thus, only one short trinucleotide, GGU, was exposed as duplicated loops 7 and 11 of the POWV DB1 and DB2, whereas the second pair of loops, i.e. 8 and 12, display sequences repeated not with each other but with loops 6 and 5 respectively, i.e. outside DB1 and DB2 (Figure S1). Additional duplicated nucleotide signals AAGG, exposed as loops, are present in POWV SL6 and SL9; moreover, the first 4 paired nucleotides of stems 6 and 9 were also identical (Figures S1 and S2). These repeated sequences were present in all POWV strains and may be biologically significant.

The region between the DB1-DB2 structures displays variability; 2 groups of TBFV were identified in respect of structural homology, one exposed a TBEV-like loop 9, whereas the second group formed a POWV-like SL9 (Figure S1). All of the TBFV displayed highly conserved TBEV-like SL11. The structures upstream of DB2 differed among the TBFV, with only SL18 conserved between all wild-type viruses, except for POWV (Figures S1 and S2). Other short 3–4-nucleotide signals exposed as loops and bulges were also observed (Figure S1).

Overall, the analysis of the entire TBFV 3′UTR revealed the presence of highly conserved duplicated DB1/DB2 structures homologous to those previously identified in the MBFV and NKV groups. Comparison of encephalitic TBEV with the relatively low-neurovirulent LGTV and LIV, or haemorrhagic disease viruses, OHFV and KFDV, revealed different secondary RNA structural patterns, possibly contributing to their distinctive pathogenicities (also discussed below).

### Duplicated 3′UTR RNA structures in the ISFV

The ISFVs include two subgroups of viruses that have been shown to replicate only in mosquitoes, or mosquito cell cultures. Thus far, they appear to reproduce either in *Aedes* species cells [7], [8] or *Culex* species cells [9], [51], [52]. *Kamiti River virus* (KRV) and *Cell Fusion Agent virus* (CFAV) 3′UTRs have two almost identical DRs [7], 67 nts long, designated R1 and R2, homologous to LRS4/LRS3 that form DB1/DB2 of the MBFV[12], [13] and TBFV (Figure S1). The R1 and R2 in KRV are separated by ∼500 nucleotides, whereas they occur as tandem repeats in the CFAV 3′UTR [17].

As no previous predictions of secondary RNA structures for the ISFV have been performed, structures in the ISFV 3′UTR were predicted using MFold as shown in Figure S3. As with TBFV, the limited sequence database of ISFV 3′UTRs excluded the application of statistical methods of analysis [22], [23]. The ISFV secondary RNA structures were observed as SLs, DB-like and Y-shaped structures. They are enumerated in the 3′→5′ direction and annotated with the features revealed by the alignments. The predicted ISFV secondary RNA structures were also validated by comparison with homologous RNA structures predicted for the 3′UTR of the distantly related MBFV and NKV, for which a variety of methods of RNA structural analysis have been used [23], [36], [49], [50], [53].

The MFold-predicted RNA structures for KRV, CFAV and *Culex flavivirus* (CxFV) are shown in Figure S3. As with other flaviviruses, a terminal 3′LSH was detected with a 3′CPN CACCG homologous to the TBFV/MBFV CACAG and NKV CUCAG/CCUAG [36]. As with other flaviviruses, the ISFV 3′CPN is localised in the apical position of the 3′LSH with the first nucleotide forming the base pair and the remaining four bases exposed as loop 1. However, in contrast to other flaviviruses, none of the ISFV showed a fold corresponding to a short SL2 in close proximity to the 3′LSH, indicating some differences in the organisation of “canonical” regions of the flavivirus promoter.

Remarkably, a significant secondary RNA structural duplication of the 3′LSH-Y1 region was observed precisely between the two “halves” of the KRV 3′UTR. The 3′CPN of the 3′LSH top loop, CACCG, was homologous to the sequence CUUCG localised in an apical position of SL16. The Y1 structure was homologous to Y5, with notably similar short signals UUU and AUG exposed at the top of the loops. These data confirm the previous suggestion that the KRV 3′UTR was formed as a result of self-duplication of a sequence of ∼600 nts [17].

The most striking feature noted for each ISFV was the unusually large number of duplicated short signals, about 3–4 nucleotides in length, exposed as top loops. Some of these signals were even tri- and quadruplicated (identically colour-coded in Figure S3). However, the exposed duplicated signals were specific for KRV, CFAV or CxFV with only a small degree of sequence homology detected between them.

The R1 and R2 of KRV are involved in the formation of 2 and 1 DB-like structures respectively that expose loops of short sequence: 3 of GAAA and 2 of CAA (Figure S3). Two of these GAAA loop sequences are coordinated by two short stems, which are identical in sequence. This conservation, in contrast to the diversity of the surrounding regions suggests a biological function.

In CFAV, the tandem R1–R2 is involved in formation of 3 DB-like structures that are not structurally homologous to those encoded by the KRV R1 and R2, although one of the KRV-like triplicated signals GAAA is exposed as CFAV loop 13 (Figure S3). The repeat GAAA is found not within the CFAV R1–R2 region as expected but in loop 3 of the Y-1. The second repeated feature within the R1–R2 tandem is an almost identical presentation of SL9 and SL12, where the duplication is not limited to the exposed short signal G(C/U)AA but involves more extended areas of ssRNA (as a bulge) and dsRNA (stem) regions (Figure S3).

Overall, there was a lack of significant structural homology between the R1 and R2 of KRV and CFAV and their conservation is therefore presumably related to a shared biological requirement for duplicated sequences.

**Figure 2:**
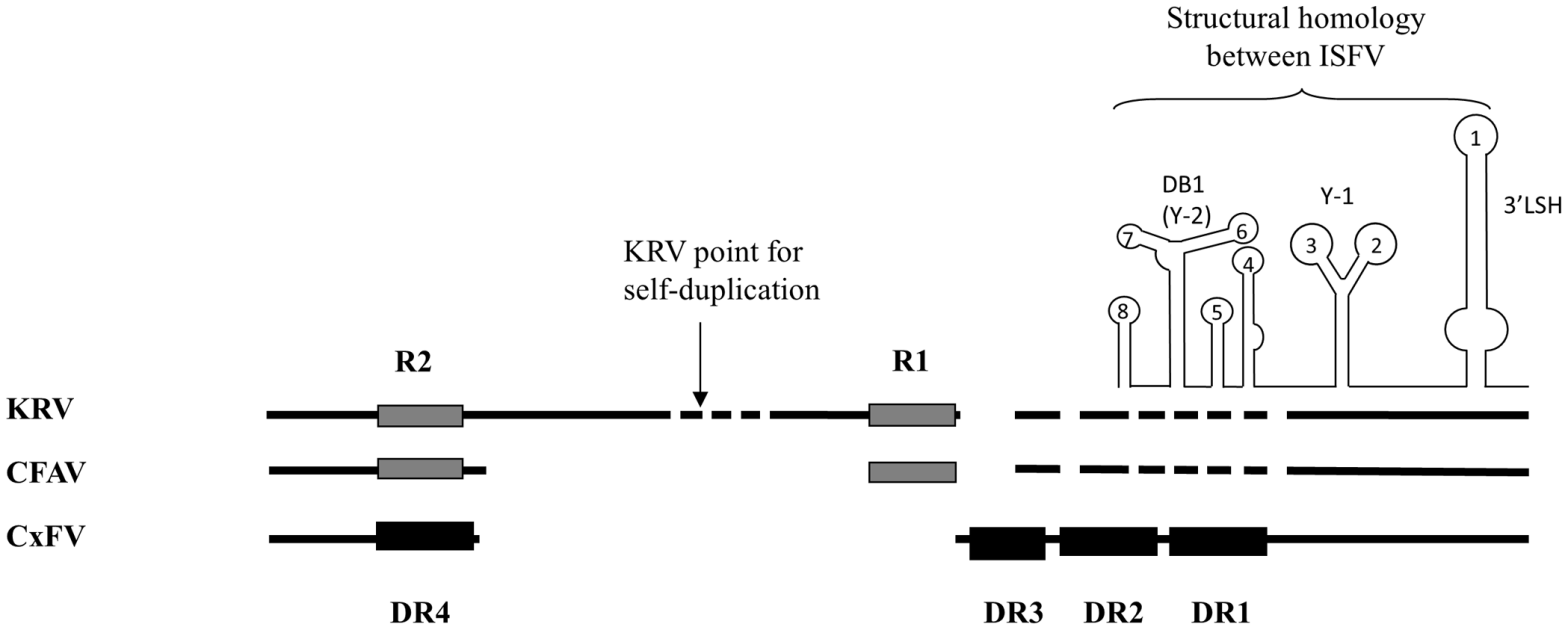
Evolution of ISFV direct repeats and RNA structures. The alignment between *Aedes*-and *Culex*-associated ISFV 3′UTRs is presented schematically based on the alignment in Figure S4A. The CxFV DRs1-4 are designated as black boxes and *Aedes*-associated R1/R2 as grey boxes; different box shadows reflect the independent origin of the CxFV DRs as discovered by the alignments in Figure S5A. The regions of very low sequence homology between *Aedes*- and *Culex*-associated ISFV are shown as dashed lines and the deletions as gaps. RNA conformations conserved between all ISFV are scheduled and specified based on the MFold-generated images in Figure S3. The RNA structures upstream of DB1 vary significantly between the ISFV and are not presented.

Numerous branched Y-shaped/DB-like structures and duplicated loop signals were the distinctive feature of the ISFV 3′UTR (Figure S3) and to understand the significance of these observations and to relate the RNA structures of KRV, CFAV and CxFV to each other, a range of alignments of the 3′UTRs of ISFV was constructed and annotated (Figure 2 and Figures S4 and S5A).

### The independent origin of *Aedes*- and *Culex*-associated DRs

The homology between the entire 3′UTR of *Aedes*-associated (KRV/CFAV) and *Culex*-associated (CxFV) ISFV was low (26–38%) but nonetheless alignment between all ISFV 3′UTRs was possible (Figure S4A). The 3′UTR of KRV and CFAV strains displayed high homology (100% and 75% respectively) although the overall 3′UTR homology between KRV and CFAV was low (27–36%) as the result of a ∼500 nts gap. Seven out of the 8 available CxFV strains displayed very close homology (91–100%) despite being isolated from geographically diverse regions of the world (USA, Japan, Mexico, China) and from 2 different vectors, i.e. *Culex pipiens* and *Culex quinquefasciatus* (complete alignment is available on request). One CxFV strain isolated from *Culex tritaeniorhynchus* (QBV) was more distantly related (55% homology). The major diversity between CxFV stains was observed in the internal hypervariable region between nucleotides 1–313. It contained long insertions/deletions, which contributed to the overall low *Culex*-specific 3′UTR homology. Two groups of the CxFV 3′UTR were identified, one closely related to the CxFV strain isolated in Japan (referred to here as CxFV J) and the second represented by QBV isolated in Vietnam.

A complication in the overall ISFV comparative alignments was short quadruplicate imperfect DRs of ∼25 nts present for CxFV (NC_008604) and homologous to R1/R2 of the KRV/CFAV group [9]. These short 25 nts-long DRs were used as an anchor to align the flanking regions (Figure S5A). This approach revealed that the CxFV quadruplicates were much longer than previously described [9]; virtually the entire region between Y1 and the stop codon is split into 4 repeats (DRs1–4) consisting of >100 nt each that also align to the KRV/CFAV R1 and R2 regions (Figure S5A). The 5 identical insertions/deletions divide this comparative alignment into two groups, *Aedes*-associated (KRV/CFAV) and *Culex*-associated (CxFV), and could reflect the result of independent duplication events based on the same 3′UTR region.

The homology between KRV/CFAV R1/R2 and CxFV DRs1–4 was calculated (Table 1) as defined in Material and Methods. The sequence identity between KRV/CFAV R1 and R2 was high (ranging between 51–69%) and a similar identity of 55–69% was observed between DRs1–4 of CxFV confirming a common origin for R1/R2 within the KRV/CFAV group and for DRs1–4 within the CxFV group. However inter-group homology between KRV/CFAV R1/R2 and CxFV DRs1–4 was low (Table 1) corresponding with the alignment in Figure S5A. For the CxFV QBV (FJ644291) the homology of DR4 to each of the DRs1–3 was lower (38–45%) in contrast to the higher homology between CxFV DRs1–3 (61–66%). The homology between *Culex*-associated ISFV DRs1–4 and *Aedes*-associated R1/R2 was low, ranging between 29–43% again supporting the concept of an independent evolutionary origin for the *Aedes* R1/R2 and *Culex* DRs2–4 duplications.

**Table 1:** The sequence identity matrix between DRs of ISFV.

Virus' DRs	KRV R2	KRV R1	CFAV R2	CFAV R1	CxFV J DR4	CxFV J DR3	CxFV J DR2	CxFV J DR1	CxFV QBV DR4	CxFV QBV DR3	CxFV QBV DR2	CxFV QBV DR1
KRV R2	ID	0.671	0.511	0.6	0.43	0.374	0.397	0.45	0.292	0.314	0.421	0.386
KRV R1	0.671	ID	0.62	0.686	0.428	0.416	0.375	0.428	0.323	0.299	0.4	0.395
CFAV R2	0.511	0.62	ID	0.562	0.37	0.379	0.344	0.368	0.322	0.289	0.392	0.338
CFAV R1	0.6	0.686	0.562	ID	0.388	0.416	0.35	0.372	0.377	0.319	0.375	0.359
CxFV J DR4	0.43	0.428	0.37	0.388	ID	0.687	0.55	0.603	0.44	0.473	0.579	0.536
CxFV J DR3	0.374	0.416	0.379	0.416	0.687	ID	0.625	0.554	0.539	0.468	0.585	0.52
CxFV J DR2	0.397	0.375	0.344	0.35	0.55	0.625	ID	0.591	0.448	0.557	0.616	0.644
CxFV J DR1	0.45	0.428	0.368	0.372	0.603	0.554	0.591	ID	0.462	0.503	0.601	0.63
CxFV QBV DR4	0.292	0.323	0.322	0.377	0.44	0.539	0.448	0.462	ID	0.375	0.451	0.455
CxFV QBV DR3	0.314	0.299	0.289	0.319	0.473	0.468	0.557	0.503	0.375	ID	0.656	0.559
CxFV QBV DR2	0.421	0.4	0.392	0.375	0.579	0.585	0.616	0.601	0.451	0.656	ID	0.609
CxFV QBV DR1	0.386	0.395	0.338	0.359	0.536	0.52	0.644	0.63	0.455	0.559	0.609	ID

The ISFV and DRs abbreviations were used as in Figure S5A. Produced with the use of Bioedit [18].

The alignment of the ISFV 3′UTR (Figure S4A) was annotated with the RNA predicted structures shown in Figure S3 revealing that the most conserved ISFV 3′UTR region includes the 3′LSH-Y-1 structures as scheduled in Figure 2. However, the upstream alignment of the 3′LSH-Y-1 was quite “patchy”, i.e. limited to a string of short homologous sequences. This correlates with the low overall homology between *Aedes*- and *Culex* associated ISFV in this region (Table 1). The predicted RNA structures overlap among different ISFV although the boundaries of each structure shift to different extents (Figure S4A). The positions of only a few loops are preserved and the sequences in these overlapping loops were not homologous even between closely related ISFV.

### The *Aedes*- and *Culex*-associated DRs originated from the primordial LRSs

As indicated previously, the 3′UTR of the primordial virus in the genus *Flavivirus* originated as a result of multiple duplications of LRSs, the most-preserved “virological fossils” that were first identified in the TBFV group [16]. The significant conservation of these duplicated sequences was explained by the slow TBFV molecular clock associated with their quiescent host, ticks, which limit active TBEV replication in nature to 1–2 times per year, in correspondence with their feeding patterns [11], [12], [16]. We previously reported homology between LRSs and short *Aedes*-associated ISFV R1/R2 [12]; here the homology between TBFV LRSs and the quite long CxFV-associated DRs2–4 was investigated.

Robust alignments of the TBFV and ISFV 3′UTR were limited to the 3′LSH region (Figure S4B). In this and other 3′UTR regions, TBFV demonstrates a greater homology to KRV sequences and conformations than it does to CFAV and CxFV, indicating a more ancient origin of KRV in comparison with CFAV and CxFV. This agrees with data showing that the codon preferences of KRV resemble those of TBFV, MBFV and NKV which infect vertebrates, whereas the codon preferences of other ISFV reflect their deeper adaptation to mosquitoes [54].

The homology between TBFV and ISFV in the region of the CxFV DR1 was limited although the LRS3 boundaries of CxFV and TBFV DR1 correspond. This alignment strengthens the previous suggestion that LRS2 has evolutionarily regressed in the TBFV group [12]. Notably, TBFV and KRV/CFAV share the hexanucleotide UUGGCA in loop 4 which is highly conserved among TBFV and NKV (Figure S1) and plausibly forms a kissing loop with SL6 present in the capsid gene [22]. However, these homologous sequences are not fully exposed as top loops for the KRV and CFAV and are not preserved in the CxFV group (Figures S3 and S4A).

**Figure 3:**
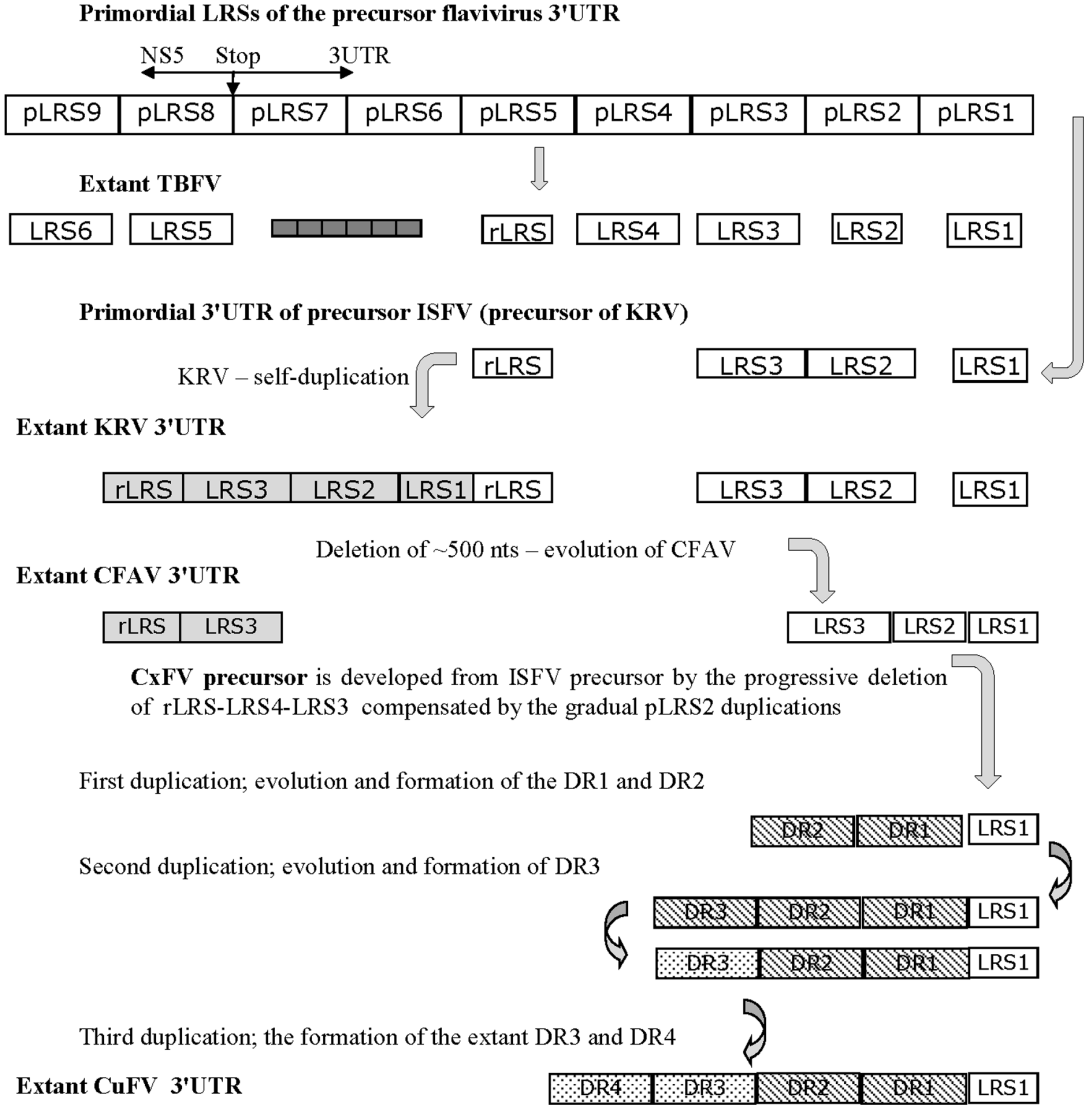
Diversification of *Aedes*- and *Culex*-associated 3′UTRs from the primordial flavivirus precursor. As previously proposed the primordial 3′UTR formed due to numerous (9 times) duplication of the C-terminal region (LRS) of the NS5^pol^ gene named the primordial LRS (pLRS) [11], [12]. The remnants of pLRSs are most highly preserved among TBFVs which also developed 6 additional short DRs (dark small grey boxes). The development of the ISFV 3′UTR may have occurred via a primordial precursor that, after significant regression, self-duplicated as described in [17]; KRV is one direct extant descendent of the ancient ISFV precursor which also gave rise to CFAV. The CxFV 3′UTR is a second direct descendant which evolved independently from the KRV/CFAV precursor by duplication of the LRS2-like region as revealed by the alignment between TBFV and ISFV in Figures S4B and S4C. Different box shading reflects the evolution of original LRSs and DRs descendants by the accumulation of nucleotide alterations.

The overall pattern of sequence and conformations upstream of the CxFV DR1 between TBFV and ISFV was different as a result of the independent duplication events within the ISFV (Figure 3). To confirm this, the TBFV LRS3–4, *Aedes*-specific R1/R2 and *Culex*-specific DRs 2–4 regions were aligned directly (Figure S4C). The alignment revealed a string of homologous regions interrupted by extensive non-homologous regions and gaps making the overall homology low (not shown). In relation to RNA structures, the CxFV DR4 and DR3 encode DB4 and DB3 respectively, with identical duplicated ssRNA signals CCA and UUA exposed as loops (Figures S3 and S4C). The CxFV DRs 2–4 encode a second pair of DB-like structures, DB1 and DB2, each with a second pair of loop duplicated signals, GUA and GCAA. This suggests that the quadruplications were unlikely to have occurred in one event but more likely occurred over 3 steps as outlined in Figure 3.

Figure S4C reveals that the DB-like structures of the TBFV and ISFV, although encoded by homologous regions of sequence, have developed from different parts of the LRSs. Thus, two major tendencies in the evolution of the 3′UTR of all ISFV are identified - the formation of numerous branched Y-shaped/DB-like structures and short duplicated signals exposed as loops. The evolutionary development of the duplicated signals appears to represent a “target” for virus evolution rather than a preservation of particular signals between major flavivirus groups and such duplications may represent examples of convergent evolution.

### Evolution of the pestivirus 3′UTR

Figure S6 shows a comparative alignment of pestivirus 3′UTRs. Previously, based on alignment of HoCV and BVDV1 3′UTRs, 2 regions, variable and conserved, were identified in the pestivirus 3′UTR [55], [56]. However, with the greater range of sequences now available, 3 distinctive regions of the 3′UTR have been identified; a proximal variable region (3′VR), a central hypervariable (3′HVR) and a distal conserved region (3′CR). The pestivirus 3′UTRs diverge significantly in the 3′VR and each virus has its own nucleotide “signature”; e.g. BVDV1 has lost the entire 3′VR.

The 3′HVR bears traces of numerous re-iterations most likely resulting from RNA polymerase stuttering on short 1–3 nt sequences. Five discrete 3′HVRs, 3′HVR1–5, represent the pestivirus primordial lineage which has since given rise to pestivirus 3′UTR diversification. The 3′HVR1 may have formed initially by the numerous reiterations of the UA-dinucleotide observed in all virus species, with the longest stretches in BVDV1, probably representing the most ancient region. The original long poly(UA) sequence was further evolved by nucleotide substitutions, deletions and insertions resulting in the formation of the UAUUGUAUA block which was subsequently duplicated several times. Four such imperfect repeats are preserved in the BVDV1 3′HVR1 and regressed sequence remnants are also observed in other pestiviruses. Further evolution included the loss of these repeats in some pestiviruses and their replacement by other sequences. For example, in the BVDV2 3′HRV1, the intercalation of AAA, followed by the further duplication of new-formed sequence blocks, resulted in the formation of two perfect DRs UGUAAAUA. Similar numerous reiterations around the tetra-nucleotide UUUA formed the 3′HVR4 (Figure S6). All pestiviruses demonstrate different levels of regressive evolution in this region, with a complete loss in the BVDV1 group. CSFV and HoCV contain unique polyU_n_C re-iterations (i.e. the 3′HVR3) which probably initiated evolution of the long hepacivirus-like 3′HVR [57], although the loss of this region in other pestivirus groups cannot be excluded.

The 3′HV5 was formed by sequences observable only in the BVDV1 group. Two short identical DRs (CUACCUCAA) are found in the 3′HV5 and downstream region (boxed in Figure S6) and appear to be part of longer, imperfect repeats. These repeats have been described for 2 other BVDV1 strains [55] and few BVDV1 strains have many deletions in this region. The sequence remnants of the 3′HVR5 in the BVDV2 group indicate that this duplicated region might have been present in the primordial pestivirus lineage.

Other notably long (60 nts) DRs have been observed in GRFPV (3′HVR2 in Figure S6) and the almost complete absence of sequence variation between DR1 and DR2 (Figure S5B) implies that this duplication occurred relatively recently. Sequence variation among pestiviruses was detected in the distal region despite it being the most conserved 3′UTR region. The robustness of the manual comparative alignment method for analysis of this region was demonstrated because it enabled the introduction of numerous gaps, making it possible to observe the natural deletion and reiteration process. Thus, alignment of the pestivirus 3′UTRs indicates that pestiviruses diverged extensively in the 3′UTR from the ancestral lineage, which may have been twice as long but regressed by sequence deletions.

### Secondary RNA structures of the pestivirus 3′UTR

Secondary RNA structures were previously identified for BVDV1 and HoCV [45], [55], [58] and are here predicted for all available pestiviruses (Figure S7). The boundaries of the stems and loops were superimposed on the pestivirus alignment (Figure S6) and structures were annotated with features revealed by the alignment.

The terminal conserved pestivirus 3′UTR region accommodates SL1 and a distal part of SL2, as observed previously [55]. Remarkably, the 3′CPN (CACAG) of the TBFV/MBFV 3′LSH was conserved in HoCV and CSFV although other pestiviruses showed single 3′CPN substitutions (Figures S6 and S7). The apical position of the 3′CPN is highly conserved, with the first two bases forming a duplex and the remaining three exposed as a loop. However, the most conserved pestivirus region is not SL1 but a 13-nt long sequence AGCACUUUAGCUG between SL1 and SL2, with the last 4 nucleotides involved in the formation of dsRNA stem 1. This region forms the top part of the SL structure at a suboptimal level of free energy (available on request) and the high degree of conservation of this structure implies an essential function yet to be experimentally determined.

The boundaries of SL2 and the position and sequence ACC(U/C)C of top loop 2 are conserved between the major pestiviruses, including HoCV, CSFV, BVDV1 and BVDV2. However, some differences were also observed; the RNDPV SL2 folded into a DB-like structure (Figure S7C) yet the sequence between SL2–SL3 still contains the sequence ACCUC. Remarkably, the BVDV1 duplicated regions DR1 and DR2 also form two homologous conformations, SL2 and rSL2 (repeated SL2), with conserved ACCUC exposed as apical loops (Figure S7G). The alignment shows that both conformations map to the SL2 region (Figure S6). However, BVDV1 DR1 and DR2 were observed only in recent isolates and are not present in the BVDV1 infectious clone (IC-BVDV1) that has been used in genetic experiments [45], [46], [59]. rSL2 may have been lost during laboratory passages - a phenomenon already described for flaviviruses [24], [26].

The 3′VR and 3′HRV1–4 fold into SLs that are very divergent in number and conformation between the pestiviruses. A notable common feature in this region is the presence of duplicated structures or short repeated linear signals of loops. Most remarkably, 2 identical 60-nt long GRFPV DRs (Figure S5B) form two pairs of repeated conformations, with duplicated sequences AAGCA and AAAUAAAUGU exposed in the apical loops (Figure S7D). Experiments with BVDV1 replicons have demonstrated that SL1 and the conserved ssRNA region between SL1/SL2 play a key role in RNA replication. Mutant viruses with deleted/modified SL1, SL2 and SL3 rescued viable viruses only if mutants contain SL1 in combination with SL2 or SL3 whereas deletions/modifications that changed SL2 or SL3 conformation, while not arresting RNA replication, reduced its rate [46], [58]. Deletions or modification of entire SL1 or SL2–SL3 failed to recover infectious viruses [59].

*In vitro* experiments with BVDV1 replicons have demonstrated that the region identified here as 3′VR-3′HRV1–5 binds a set of cellular proteins, among them a complex of nuclear factors associated with dsRNA (NFAR) proteins. These proteins also interact with the hairpin in the 5′UTR thus enabling a protein bridge between the 5′ and 3′ UTRs. Such a cyclisation of the pestivirus genome, with no direct RNA-RNA contact between 3′UTR and 5′UTR but mediated by the NFAR complex, was proposed to regulate viral translation and replication. It was suggested that this acts as a safety-lock system to prevent collisions between translation and replication complexes moving in opposite directions [45], [46]. As NFAR proteins are normally involved in regulation of transcription of genes with encoding antiviral function, such as interleukin 2, PKR and others, we suggest an alternate explanation that the pestivirus enhancer could act to sequester NFAR proteins to antagonise cellular antiviral defences. Duplication of enhancer structures would clearly be advantageous in this respect.

### Biological significance of duplicated RNA sequences and conformations in structural-functional organization of flavivirus and pestivirus 3′UTRs

Despite significant sequence and length divergence, the structure-function organization of the 3′UTR for pestiviruses and flaviviruses share common features. Both genera display the highly conserved terminal hairpin-like SL1. Flaviviruses and some pestiviruses display 3′CPN in a highly conserved apical position of the SL1 and deletion of the SL1/3′LSH abolishes pestivirus/flavivirus infectivity via reduced RNA synthesis, highlighting its role as an essential region for viral RNA synthesis.

Flaviviruses in each of the 4 ecological groups display different levels of structural conservation upstream of the SL1 [16]. SL2 is conserved between TBFV, MBFV and NKV [12] but was not observed in the ISFV (Figure S3) whereas Y-1 is present in the NKV [36], TBFV and ISFV (Figure 1, 3 and Figures S1 and S3) but had regressed in the MBFV group [12], [13] to be functionally replaced by an upstream DB1. Deletion of DB1 or DB2 but not both, retains MBFV viability [38], [40], [41] highlighting their shared role as promoter/enhancer elements in virus replication while the deletion of both DB1 and DB2 in TBFV does not prevent the recovery of infectious virus [26] implying a less critical enhancer-only function (Figure S2). Pestiviruses share SL2, conserved in the conformation and sequence of the top loop (Figures S6 and S7). However, deletion/modification of this structure does not abolish virus infectivity suggesting, in this respect, equivalence to the enhancer region of the flaviviruses. However, the pestivirus SL1 cannot function as a promoter in isolation and requires either SL2 or the upstream highly divergent SL3 region [46], [58], [59], a scenario similar to the MBFV group.

The alignment of pestiviruses revealed that non-cytopathic PV1 contained numerous deletions within the conserved part of the SL1-SL2 region [46], [58], [59] consistent with the findings in flaviviruses, whose enhancers (DB1 and DB2) are highly conserved yet redundant elements [34], [35], [39]. As with flaviviruses, conservation of these elements reflects their role in pestivirus circulation in their natural habitat rather than in cell culture.

Other similarities in terms of flaviviruses and pestivirus structural organization are the presence of long and short DRs, particularly in the internal 3′UTR regions, significantly divergent even among closely related viruses. In each group, duplicated sequences are exposed as linear signals in top loops, with the length and identity of each sequence specific only for a distinct virus group adapted to a particular host.

The molecular mechanism of RNA duplications/deletions is common to all RNA viruses, a property of the RdRp [19]. Independent convergent selection of duplicated sequences for each of the diverse ecological sub-groups of *Flavivirus* and *Pestivirus* genera suggests a biological significance for sequence and structural duplications as viruses adapt to their hosts. Numerous repeated sequences were also detected in the 3′UTR of alphaviruses although their structures and functions remained undetermined [60]. The precise function of duplicated signals is unclear but it is possible that they interact with proteins active as dimers/oligomers thus increasing the replication capacity in particular cell types, vertebrate, invertebrate or both. In addition, duplicated structures might interfere with host antivirus defence by being more efficient in sequestering key innate immune proteins from the cellular pool.

A third possibility is linked to a subgenomic flavivirus RNA (sfRNA), an almost intact 3′UTR region generated as the product of 5′-3′ XRN1 RNase processing of virus genome RNA during replication of TBFV and MBFV [47]. The presence of sfRNA correlates with pathogenicity in mammalian cells (48) and interferes with RNA silencing suppressor pathways in both mammalian and mosquito cells [61]. The 3′UTR of flaviviruses contains a large number of hairpin-like structures (Figures S1 and S3), possible targets for a Dicer [62], a cytoplasmic RNase that would cleave dsRNA regions to generate virus miRNAs as a countermeasure of cellular siRNA. In this case, the duplication of stems could increase the pool of flavivirus anti-siRNA. Indeed, the cleavage of sfRNA by Dicer has been demonstrated [63] and some duplicated hairpins were observed for flavivirus and pestivirus 3′UTRs (Figures S1 and S3 and S7). This suggests that the dsRNA regions of repeated stems might be biologically significant as a source of anti-siRNA. However, the accumulation of an intact sfRNA at the end of the virus replication cycle [47] does not fully support this suggestion. On the other hand, the numerous repeated ssRNA signals (loops and bulges) in the 3′UTR may interact directly with miRNA and siRNA; indeed both miRNA and siRNA were suppressed in vertebrate and mosquito cells by sfRNA [63].

The normal pathway for the interaction of siRNA with target mRNA includes the assembly of RISC (RNA-stimulated silencing complex) [62]. For flaviviruses, the conglomerate of SLs with RISC proteins might protect sfRNA from a complete degradation, in contrast to pestiviruses that do not produce sfRNA [47] perhaps because they have a shorter 3′UTR with fewer duplicated signals (Figure S7). The ability of flavivirus sfRNA to act as an interferon antagonist [64] implies the formation of additional RNA-protein complexes although these two pathways are probably linked [63]. Both functions may be possible via the interaction of siRNA/ISG (interferon-stimulated gene) proteins with duplicated signals of the 3′UTR diverting the immune response in mammalian and mosquito cells early in the replication cycle but the accumulation of non-processed protein aggregates triggers apoptosis and cpe in mammalian cells at later time points.

This hypothesis also explains the differences detected between RNA structures of OHFV and AHFV (Figure 1) as a group of viruses that cause haemorrhagic disease and TBEV, as a group of neurotropic viruses. Viral HFs develop following unlimited virus spread through the host as the result of the ability of the virus to supress early host innate immunity. This eventually leads to a “cytokine storm” [65], accompanied by massive influx of vasoactive molecules that disrupt homeostasis and cause haemorrhage. At this stage of our knowledge it cannot be excluded that OHFV- and AHFV-specific RNA structures (Figure S1) supress (attenuate) some ISG-related pathways activated during TBEV infections.

Insect immunity is increasingly recognised as inhibitory RNA silencing [66] and prominent SLs of sfRNA could act as an effective decoy for insect siRNA. Analysis of the ISFV group showed a level of duplicated short signals that considerably exceeded those in other flavivirus or pestivirus groups as would be expected of an insect immunity-driven selection pressure. Paralleling the use of “molecular sponges” to supress cellular miRNA [67], duplicated signals of 3–6- nucleotides of ISFV might act to sequester miRNA/ISG-proteins to overcome pathways involved in insect innate immunity [66]. Such an explanation would be consistent with an enhancer region that is redundant for laboratory-maintained viruses yet highly conserved among wild virus species.

In conclusion, numerous duplicated RNA structures were identified in the enhancer regions of the 3′UTR of distantly related flaviviruses and pestiviruses. Analysis of virus sequences indicated that duplicated homologous RNA structures have emerged independently on numerous occasions in different virus groups of both genera as the result of convergent evolution. Identifiable as redundant for laboratory maintained viruses, these structures are highly conserved implying their significant biological role, rather likely as enhancers of virus replication and/or antagonists of cellular antiviral innate immunity. The latter possibly play a role in direct contact between virus and host molecular elements, acting as adaptors of virus replication cycles to the molecular machinery of the particular host, with its mi/siRNA and thus determining virus host range and its pathogenetic characteristics.

## Supporting Information

Figure S1 **Predicted secondary RNA structures of TBFV 3′UTR.** Images were produced for A) TBEV, B) LGTV, C) OHFV, D, E) LIV and F) POWV by MFold using MDBP = 80 and annotated with LRSs, 3′CYCL (brown line), a 3′CPN (dark pink background) and a conserved hexanucleotide (red background). Viruses and corresponding accession numbers are indicated. Promoter is enclosed in a red-lined box. The colour/box codes on the left top corner designate RNA conformations of TBFV that are conserved throughout the *Flavivirus* genus. Identical sequences of duplicated RNA conformations DB1 and DB2 and other repeated sequences are indicated by identical colours.
(PDF)

Figure S2 **Alignment of TBFV 3′UTR (to view with magnification 130–200%).** Viruses are identified by abbreviated names and accession numbers. The RNA conformations (SLs and Y-shaped) and top loop regions are outlined by square and semi-oval brackets respectively and are enumerated in correspondence with Figure 1. The loops of individual viruses not conserved between TBFV are enclosed in ovals. The boundaries of LRSs, short DRs, V3′UTR and C3′UTR are outlined by vertical arrows. The boundaries between the viable and the non-viable engineered viruses [26], [43] corresponding to the promoter and enhancer parts of the 3′UTR are indicated. Alignment with a complete list of 3′UTR sequences is available on request.
(PDF)

Figure S3 **Predicted RNA structures of the ISFV.** Images for 3′UTR for A) KRV, B) CFAV and C, D) CxFV were produced using the MFold MDBP = 80 and annotated with features of Figure S4. The SLs and Y-shaped structures are enumerated. The KRV/CFAV R1/R2 are outlined by red lines and boundaries of CxFV-DRS2-4 are indicated. The duplicated sequences are indicated by the identical color code on each image. A putative pseudoknot for KRV and kissing loops for the CxFV QBV are indicated.
(PDF)

Figure S4 **ISFV 3′UTR alignments (to view with magnification 130–200%).** Viruses are identified by the abbreviated names and accession numbers. The R1 and R2 correspond to 67-nt long DRs of the KRV and CFAV identified in [7]. **A**. The alignment between 3′UTR of *Aedes*- (KRV and CFAV, black letters) and of *Culex*-associated (red letters) ISFV annotated with RNA conformations (oval brackets) displayed in Figure S3. The boundaries of extended CxFV DR1-DR4 are indicated in blue arrowed lines. **B**. Alignment between TBFV and ISFV annotated with secondary RNA structures as scheduled in Figures S1 and S3 in the region of the CxFV 5′DR1-3′LSH. Red semi-oval brackets specify loops with sequences similar between TBFV and ISFV. The regions of CxFV DR1 and TBFV LRS2-LRS3 are indicated. **C**. Alignment between TBFV LRS3–4 and ISFV DRs1–4/R1–2. The loop sequences of TBFV DB1 and DB2 are enclosed in ovals. Solid lines, with appropriate colour code, outline the positions of the DB-like and SLs encoded by the repeated regions; the arrows indicate the extension of the DB-like structure outside the alignment region shown.
(PDF)

Figure S5 **Convergent duplications of flavivirus and pestivirus sequences in the 3′UTR.** A. Independent origin of *Aedes* (KRV/CFAV) R1/R2 (red-box) and *Culex* DRI-DR4 (black-box) duplicated ISFV sequences. Virus 3′UTR sequences are enumerated from the stop codon of the single ORF. The CxFV 3′LSH and 3′Y-1 structures are mapped to the alignment. The *Culex*- and *Aedes*-specific gaps are enclosed in grey areas. The full-length of the *Culex-* associated ISFV 3′UTR is presented whilst only aligned fragments of KRV and CFAV 3′UTR are shown. B. Sequence identity of GRFPV duplications.
(PDF)

Figure S6 **3′UTR alignment of pestiviruses (to view with magnification 130–200%).** Viruses are designated by accession numbers and abbreviated names. The variable (3′VR and 3′HVR1–5) and the conserved (3′CR) regions are indicated. The DRs are shown by double-headed arrows or boxed, with an appropriate colour code. The RNA conformations (SLs and Y-shaped) and the top loop regions are outlined by square and semi-oval brackets respectively and are enumerated as in Figure S7.
(PDF)

Figure S7 **Predicted secondary RNA structures for the pestiviruses.** Images were produced by MFold using MDBP = 60 for **A**) HoCV; **B**) CSFV; **C**) RNDPV; **D**) GRFPV; **E**) BDV; **F**) BDVD2; **G**)BVDV1. Viruses are identified by the abbreviated names and accession numbers. The SLs are numerated and annotated with the conserved 3′CPN (highlighted in red), conserved loop 2 sequences (highlighted in bright green) and conserved ssRNA region (blue line). The DRs of each pestivirus are highlighted in identical colours.
(PDF)

Table S1 **List of abbreviations.**
(PDF)
