# Supplementary material for: Molecular Archaeology of Flaviviridae Untranslated Regions: Duplicated RNA Structures in the Replication Enhancer of Flaviviruses and Pestiviruses Emerged via Convergent Evolution
Source: PLoS One. 2014 Mar 19;9(3):e92056. doi: 10.1371/journal.pone.0092056 (PMC3960163; doi:10.1371/journal.pone.0092056)

Figure S1. Predicted secondary RNA structures of TBFV 3'UTR.

Figure S1A
TBEV Sib
AB049399

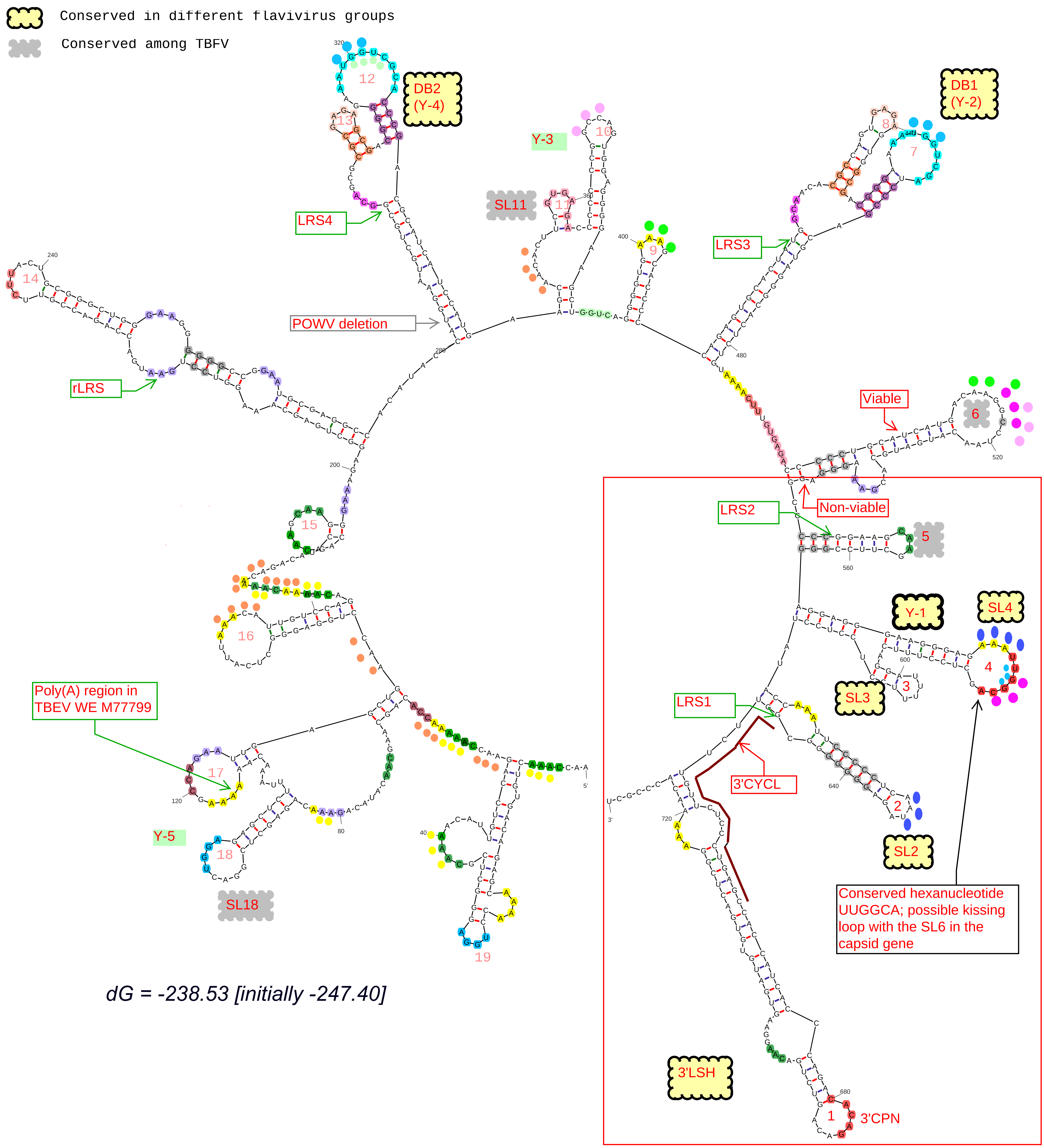

dG = -238.53 [initially -247.40] TBEV IR99-2m7 Sib AB049399 80mdbp

Figure S1B
LGTV
NC_003690

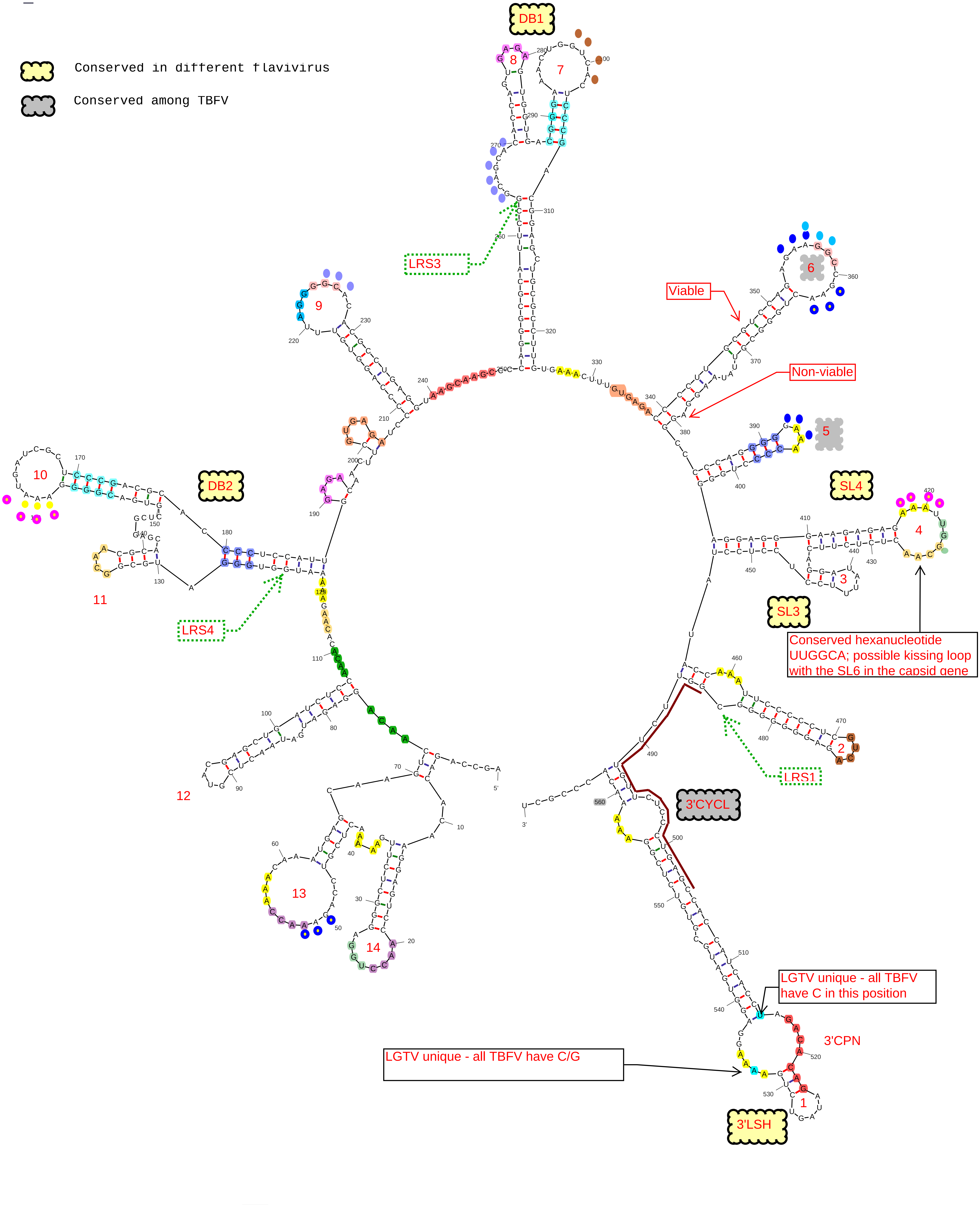

dG = -202.35 [initially -211.90]

Figure S1C
OHFV
AB062063

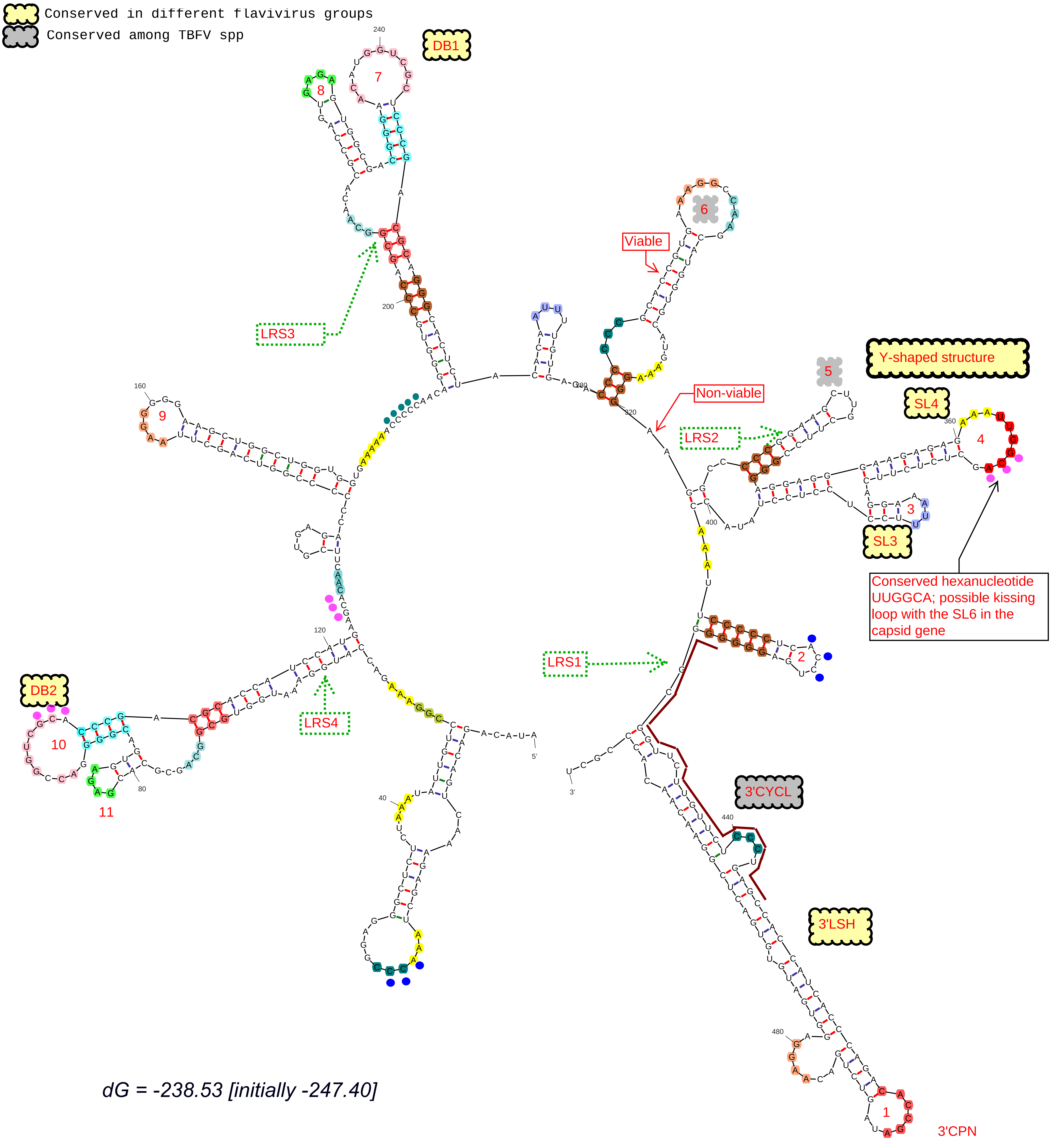

Figure S1D
LIV
Y07863

Conserved in different flavivirus groups
Conserved among TBFV spp

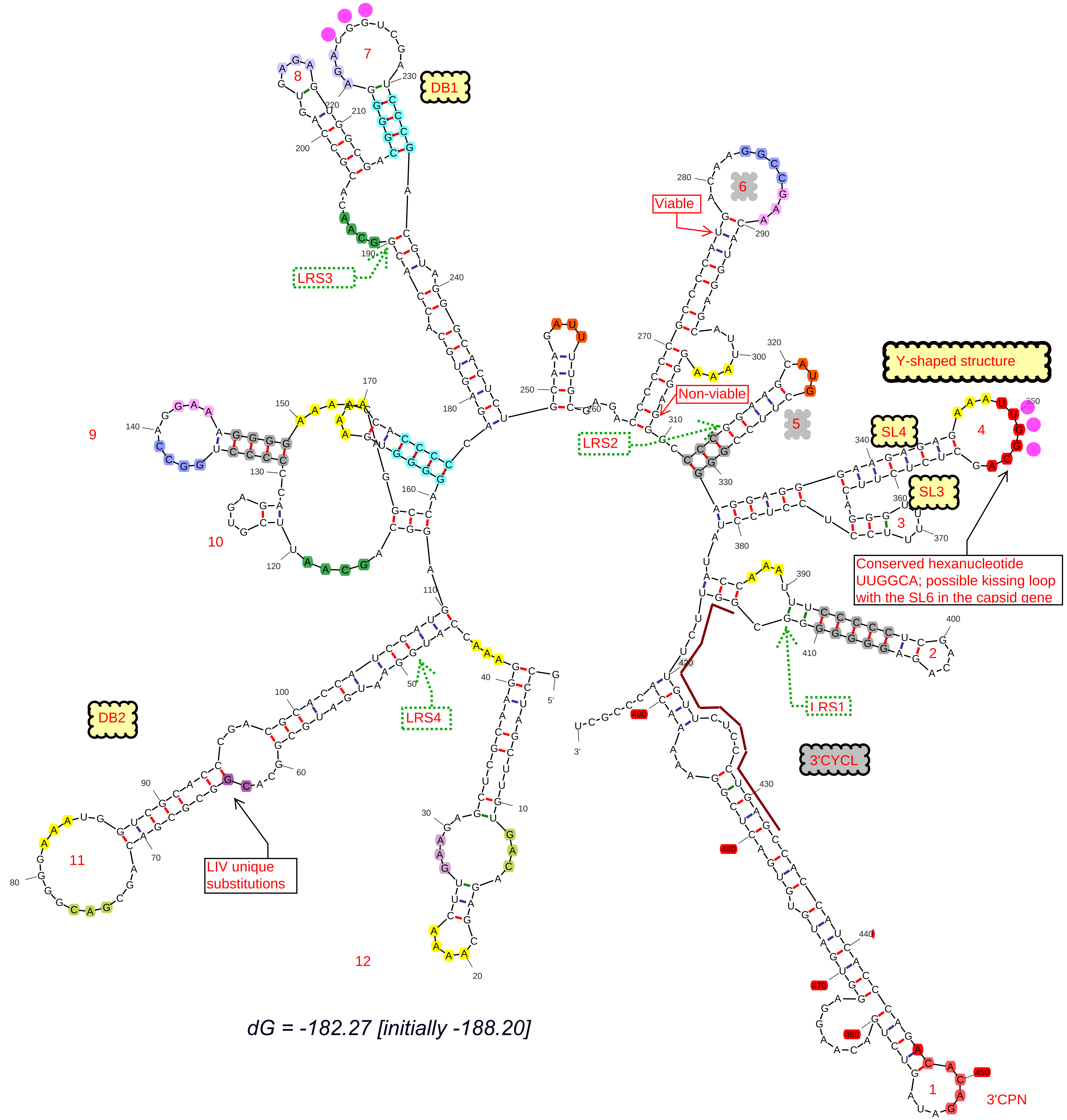

Figure S1E
LIV
$C_{63}G_{64} \rightarrow G_{63}C_{64}$
Y07863

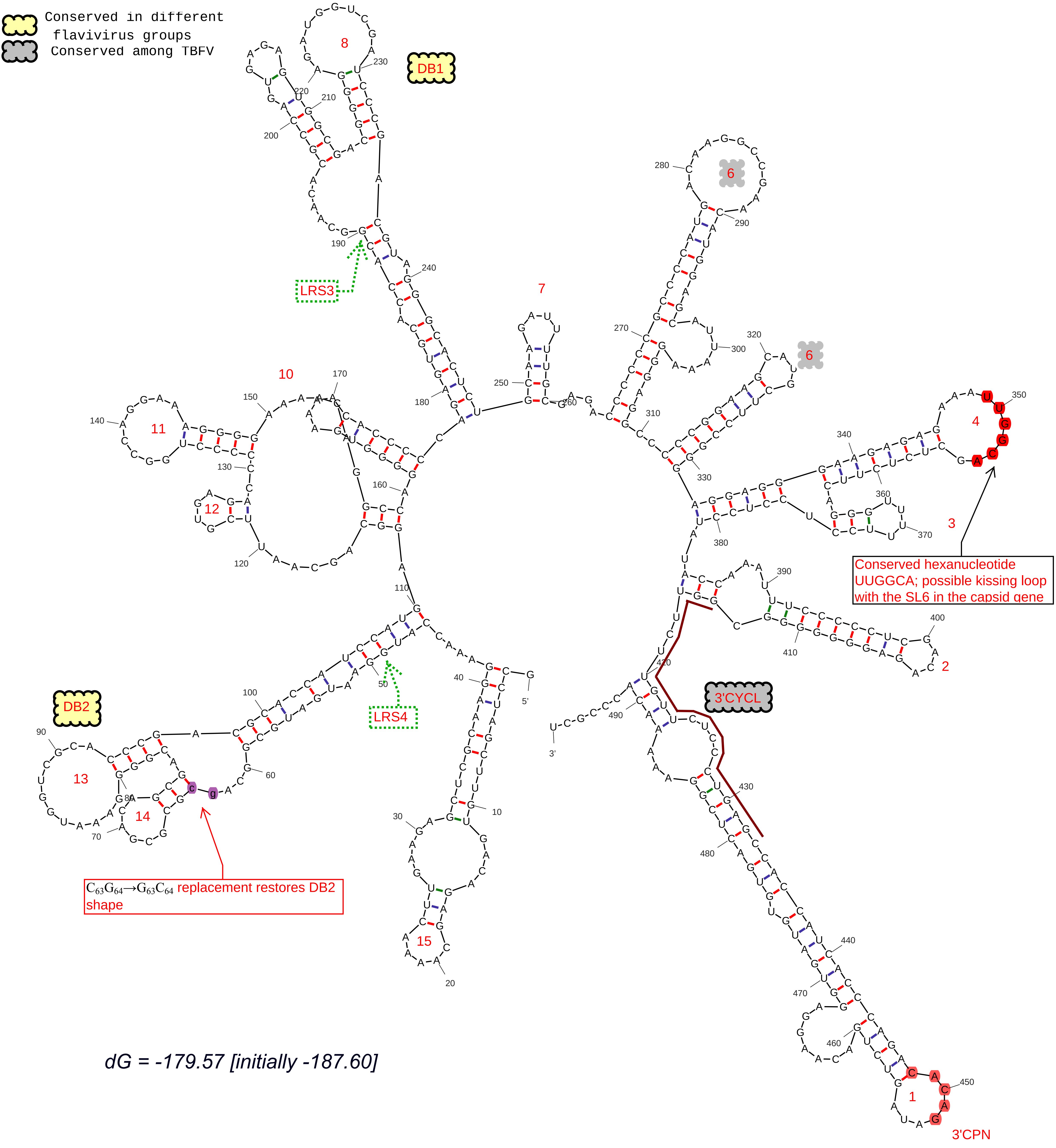

Figure S1F
POWV
NC_003687

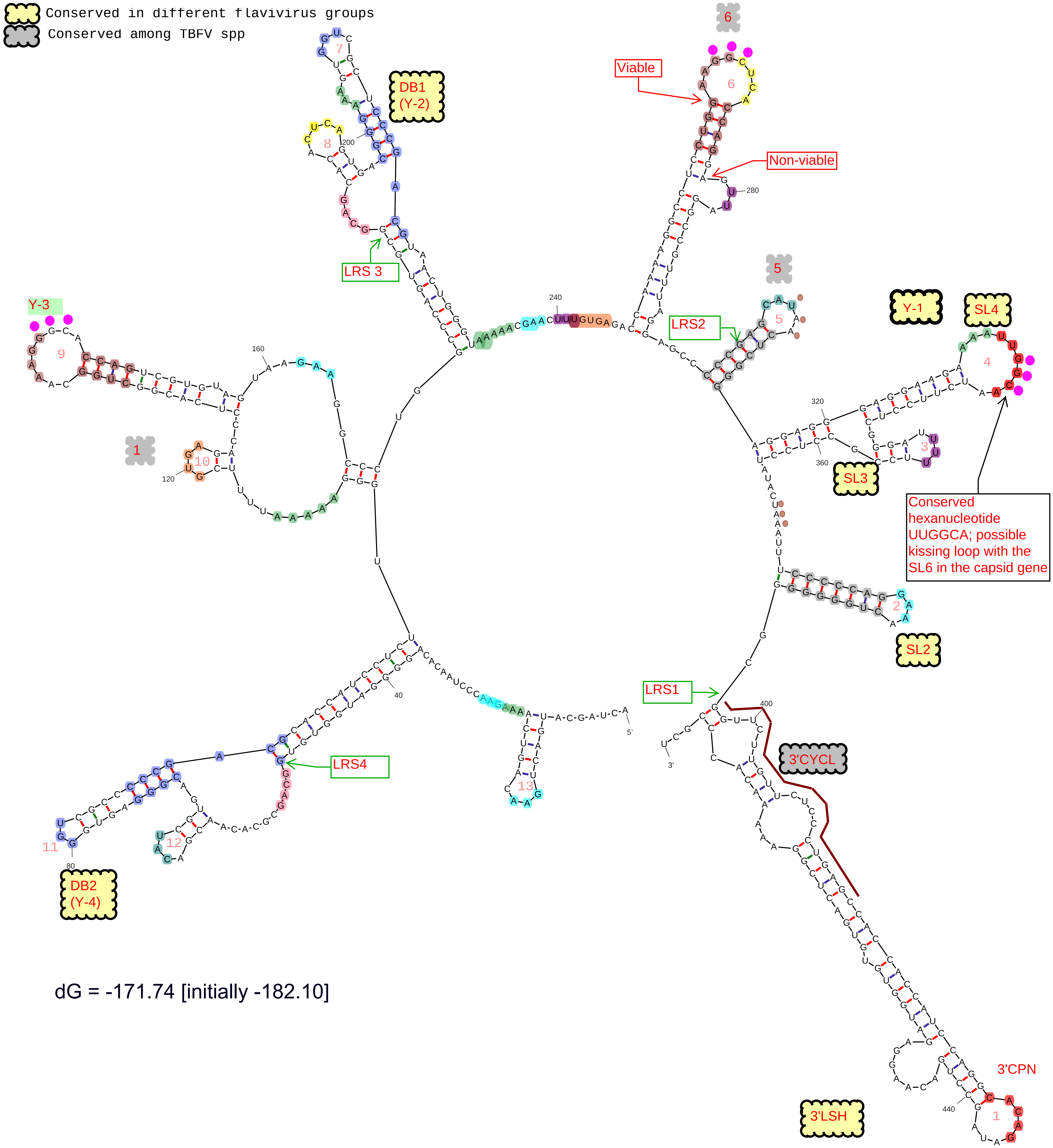

Supplement: Figure S1 — Predicted secondary RNA structures of TBFV 3′UTR. Images were produced for A) TBEV, B) LGTV, C) OHFV, D, E) LIV and F) POWV by MFold using MDBP = 80 and annotated with LRSs, 3′CYCL (brown line), a 3′CPN (dark pink background) and a conserved hexanucleotide (red background). Viruses and corresponding accession numbers are indicated. Promoter is enclosed in a red-lined box. The colour/box codes on the left top corner designate RNA conformations of TBFV that are conserved throughout the Flavivirus genus. Identical sequences of duplicated RNA conformations DB1 and DB2 and other repeated sequences are indicated by identical colours. (PDF) [file pone.0092056.s001.pdf]
