# Supplementary figures and images for: Molecular Archaeology of Flaviviridae Untranslated Regions: Duplicated RNA Structures in the Replication Enhancer of Flaviviruses and Pestiviruses Emerged via Convergent Evolution
Source: PLoS One. 2014 Mar 19;9(3):e92056. doi: 10.1371/journal.pone.0092056 (PMC3960163; doi:10.1371/journal.pone.0092056)

Figure S2. Alignment of TBFV 3'UTR.

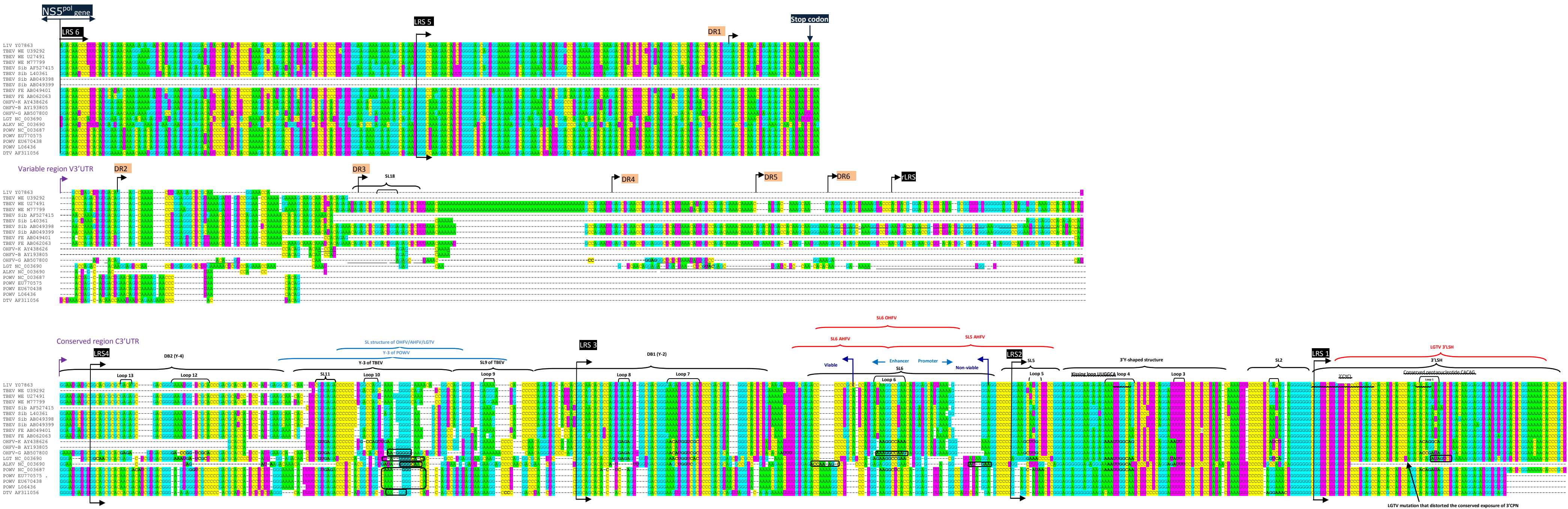

Supplement: Figure S2 — Alignment of TBFV 3′UTR (to view with magnification 130–200%). Viruses are identified by abbreviated names and accession numbers. The RNA conformations (SLs and Y-shaped) and top loop regions are outlined by square and semi-oval brackets respectively and are enumerated in correspondence with Figure 1. The loops of individual viruses not conserved between TBFV are enclosed in ovals. The boundaries of LRSs, short DRs, V3′UTR and C3′UTR are outlined by vertical arrows. The boundaries between the viable and the non-viable engineered viruses [26], [43] corresponding to the promoter and enhancer parts of the 3′UTR are indicated. Alignment with a complete list of 3′UTR sequences is available on request. (PDF) [file pone.0092056.s002.pdf]

Figure S6. 3'UTR alignment of pestiviruses.

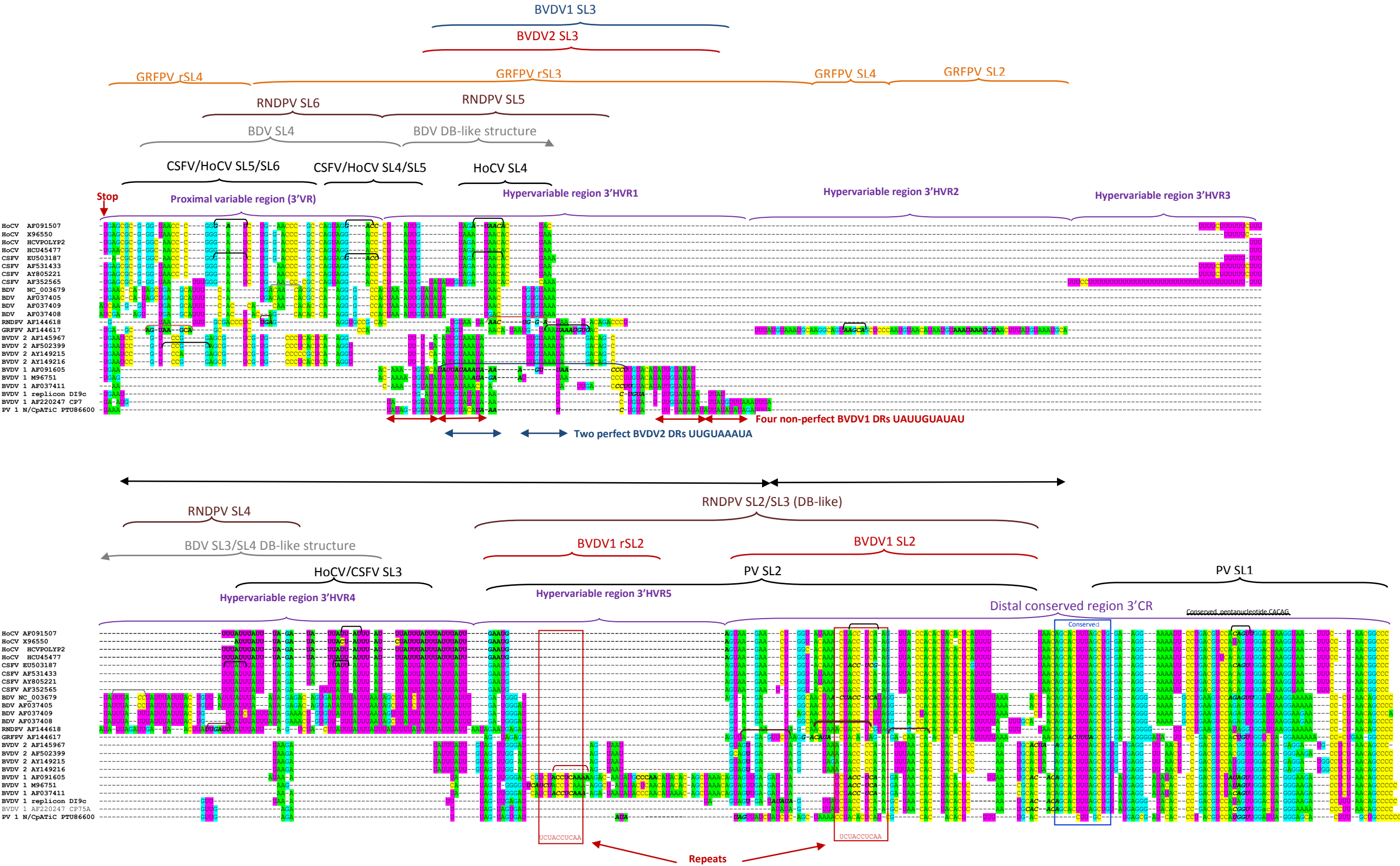

Supplement: Figure S6 — 3′UTR alignment of pestiviruses (to view with magnification 130–200%). Viruses are designated by accession numbers and abbreviated names. The variable (3′VR and 3′HVR1–5) and the conserved (3′CR) regions are indicated. The DRs are shown by double-headed arrows or boxed, with an appropriate colour code. The RNA conformations (SLs and Y-shaped) and the top loop regions are outlined by square and semi-oval brackets respectively and are enumerated as in Figure S7. (PDF) [file pone.0092056.s006.pdf]
