# Supplementary material for: Molecular Archaeology of Flaviviridae Untranslated Regions: Duplicated RNA Structures in the Replication Enhancer of Flaviviruses and Pestiviruses Emerged via Convergent Evolution
Source: PLoS One. 2014 Mar 19;9(3):e92056. doi: 10.1371/journal.pone.0092056 (PMC3960163; doi:10.1371/journal.pone.0092056)
Supplement: Figure S3 — Predicted RNA structures of the ISFV. Images for 3′UTR for A) KRV, B) CFAV and C, D) CxFV were produced using the MFold MDBP = 80 and annotated with features of Figure S4. The SLs and Y-shaped structures are enumerated. The KRV/CFAV R1/R2 are outlined by red lines and boundaries of CxFV-DRS2-4 are indicated. The duplicated sequences are indicated by the identical color code on each image. A putative pseudoknot for KRV and kissing loops for the CxFV QBV are indicated. (PDF) [file pone.0092056.s003.pdf]

Figure S3. Predicted RNA structures of the ISFV.

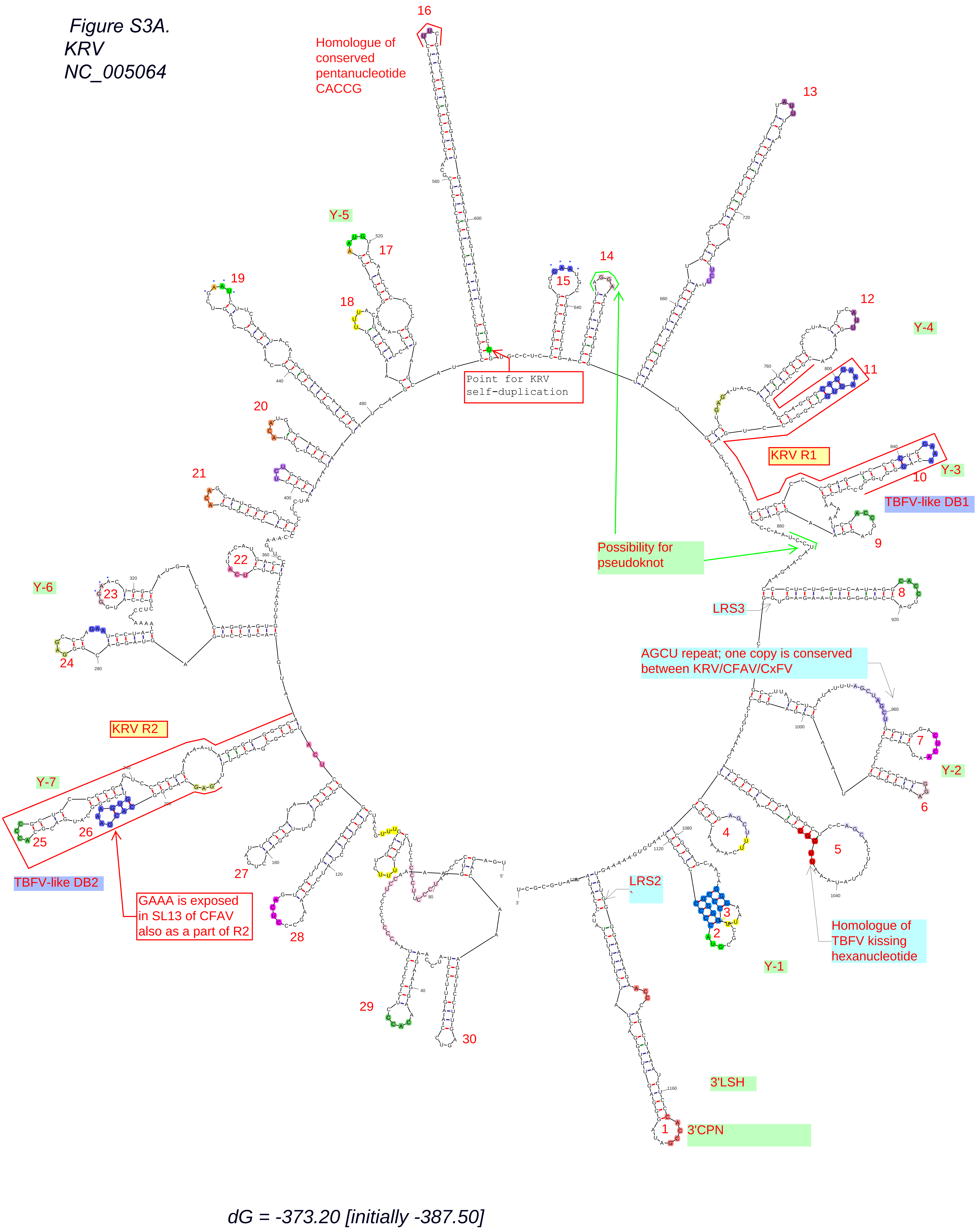

Figure S3B.
CFAV
NC_001564

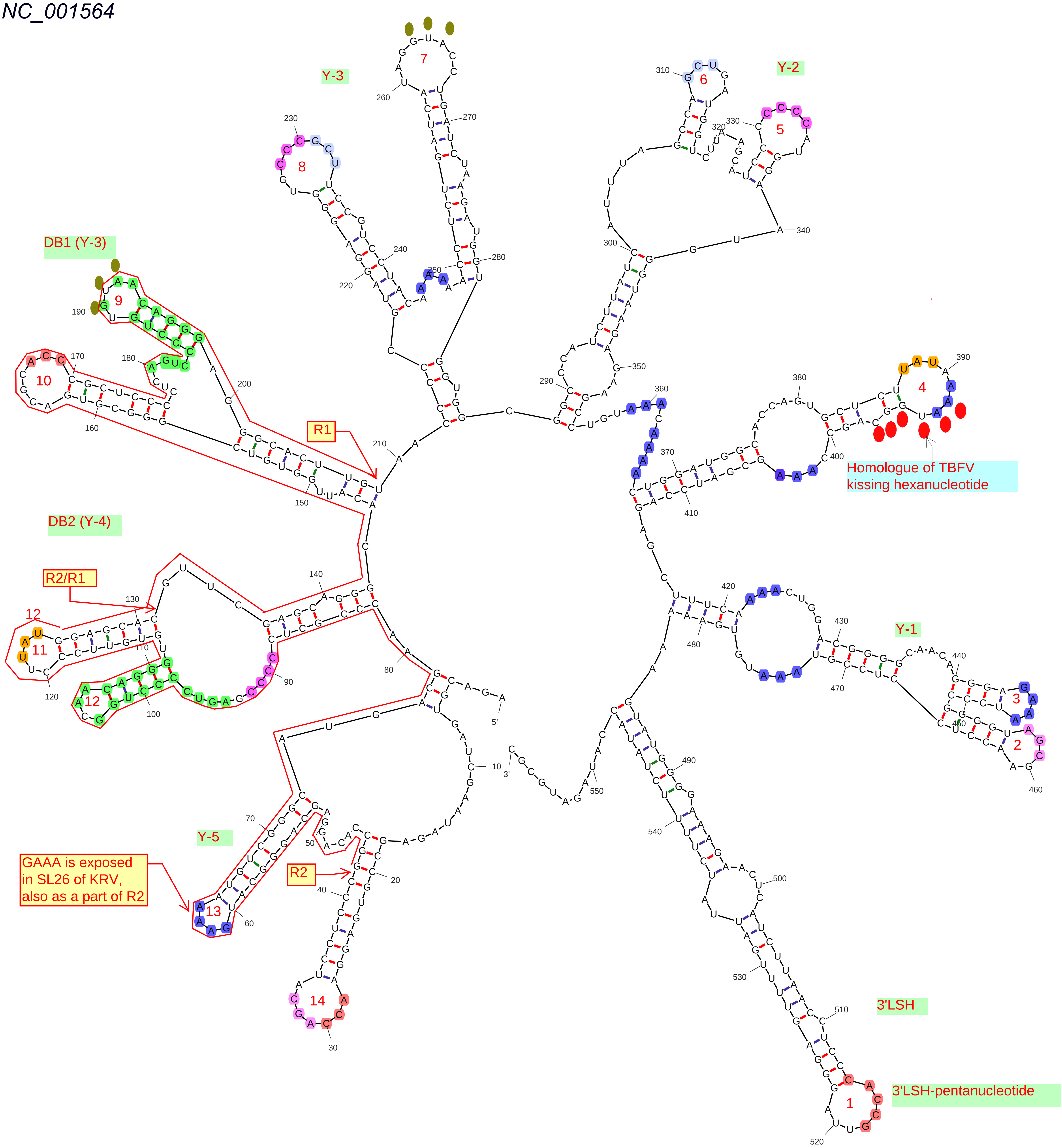

dG = -162.86 [initially -177.10]

Figure S3C
CxFV J
NC_008604

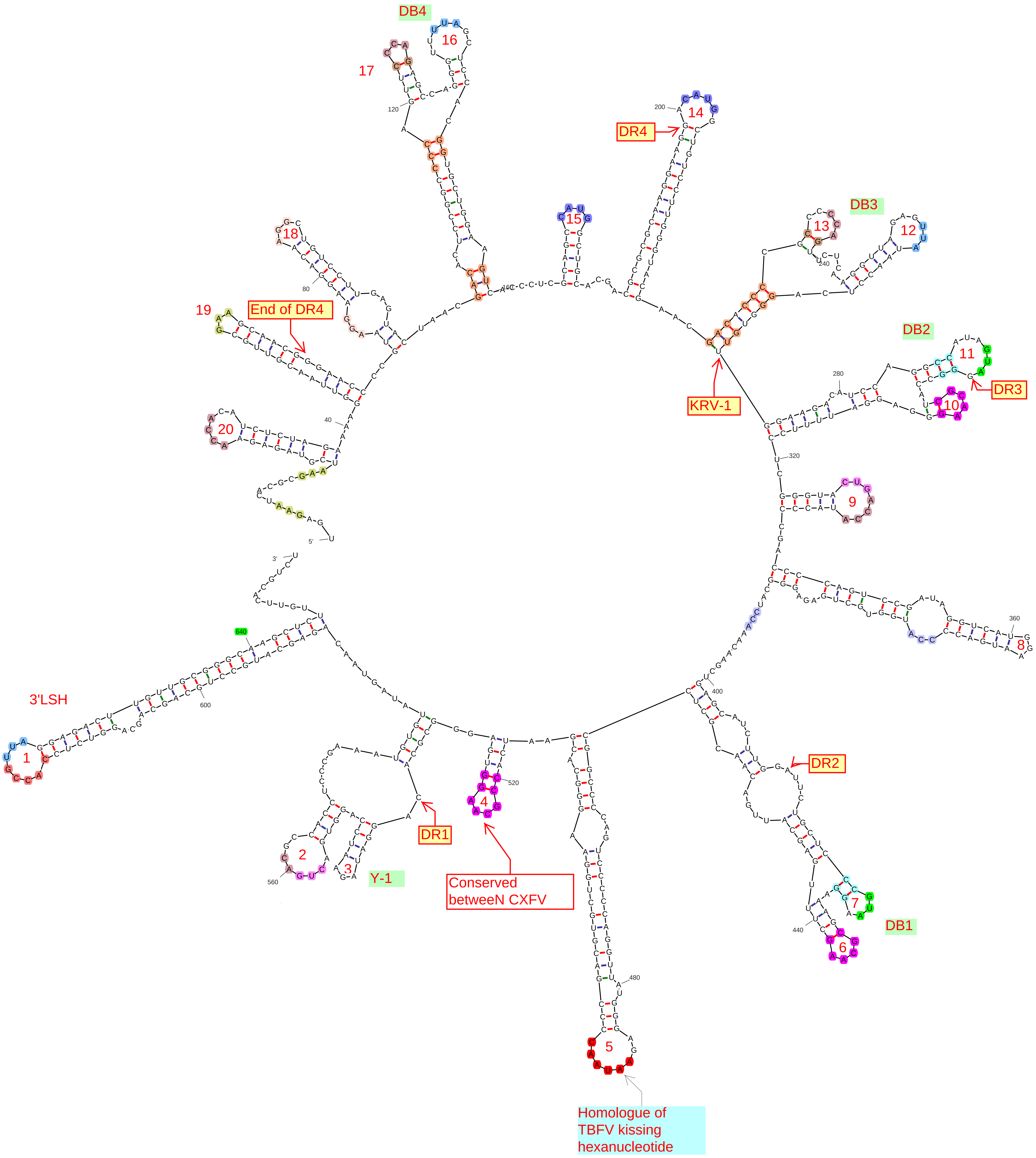

dG = -216.41 [initially -226.80]

Figure S3D.
CxFV QBV
FJ644291

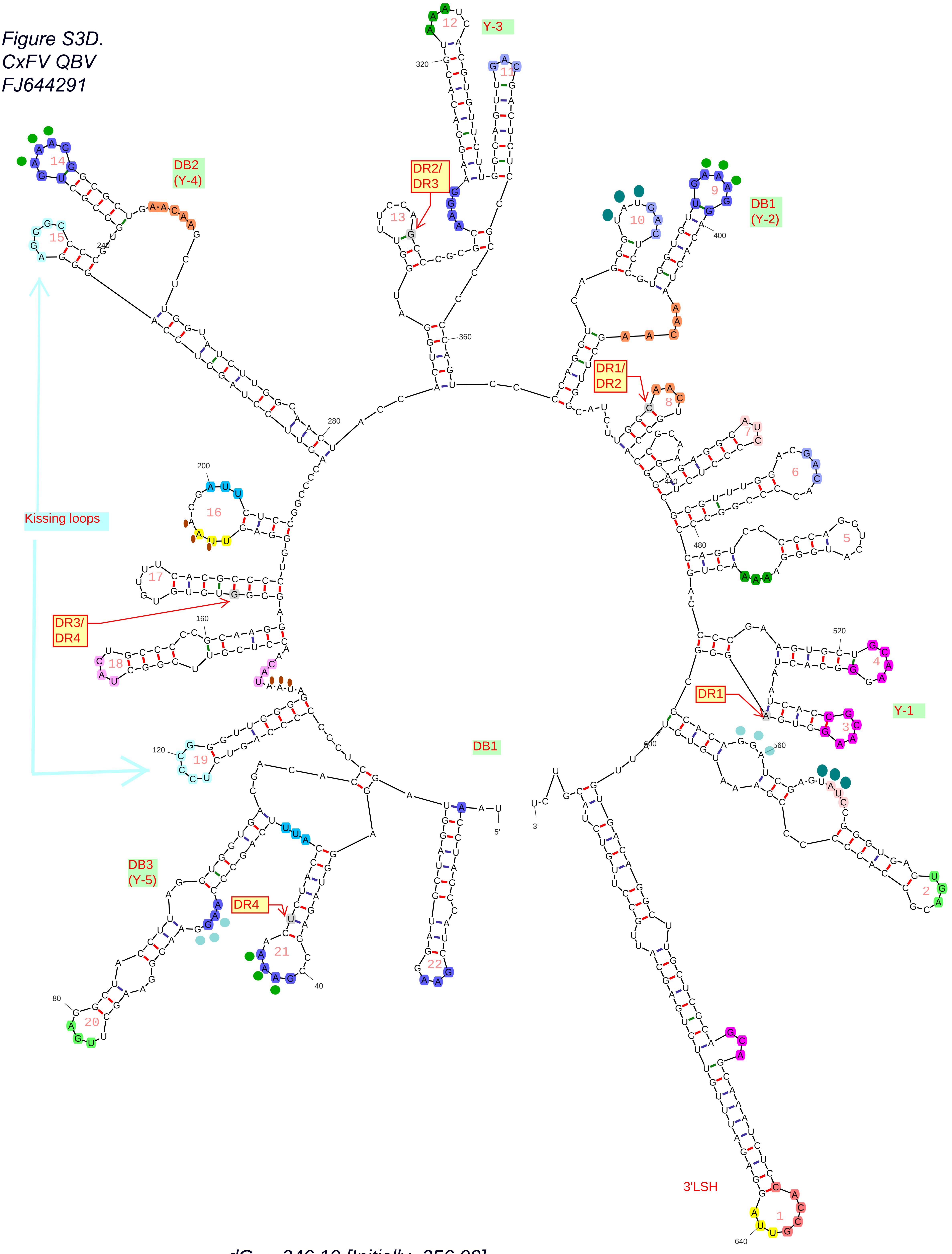

dG = -246.19 [Initially -256.00]
