# Supplementary material for: Molecular Archaeology of Flaviviridae Untranslated Regions: Duplicated RNA Structures in the Replication Enhancer of Flaviviruses and Pestiviruses Emerged via Convergent Evolution
Source: PLoS One. 2014 Mar 19;9(3):e92056. doi: 10.1371/journal.pone.0092056 (PMC3960163; doi:10.1371/journal.pone.0092056)

Figure S4. ISFV 3'UTR alignments.

Figure S4A. Alignment between 3'UTR of *Aedes*- and of *Culex*-associated ISFV.

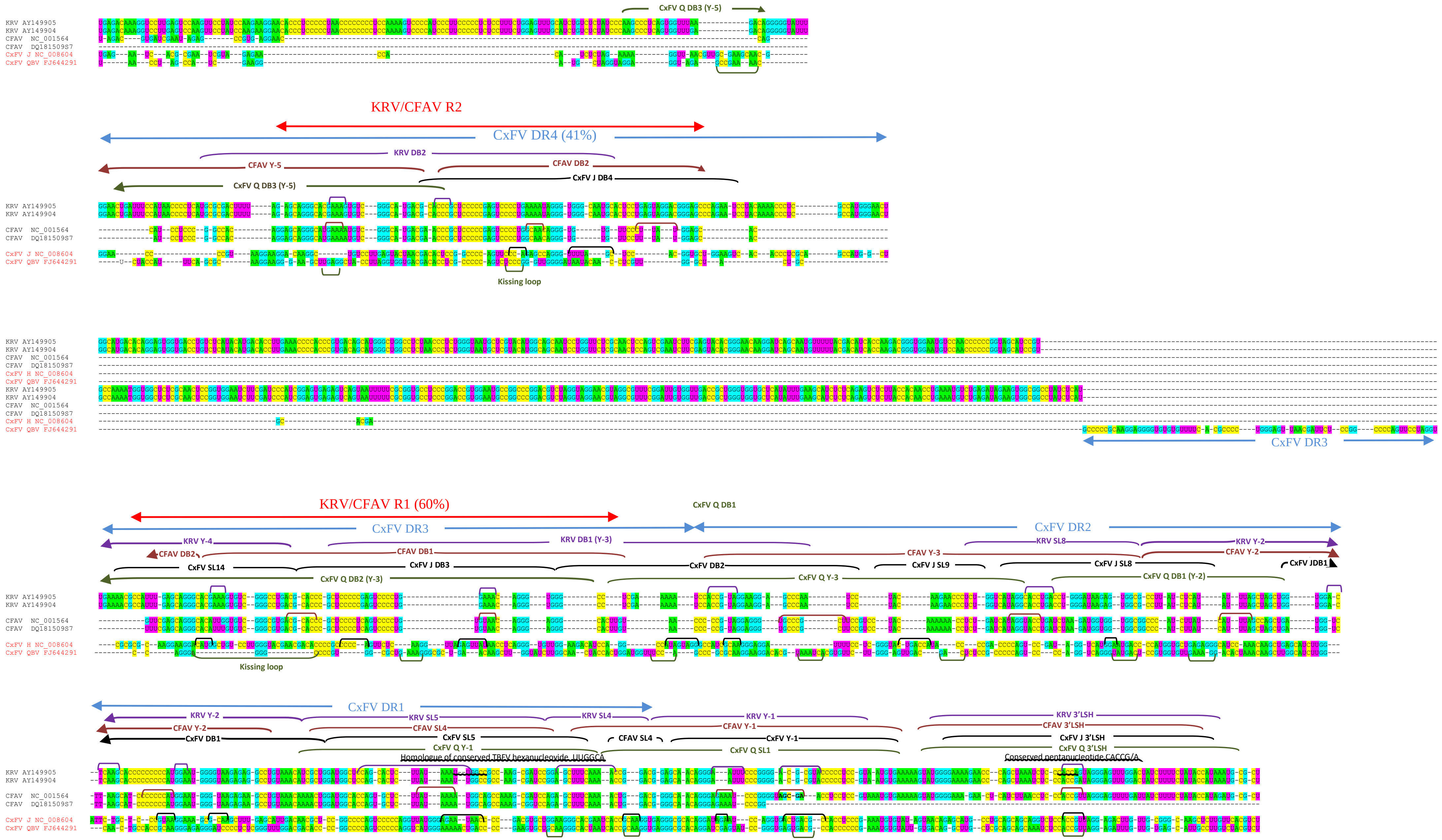

Figure S4B. Alignment between 3'UTR of TBFV and ISFV

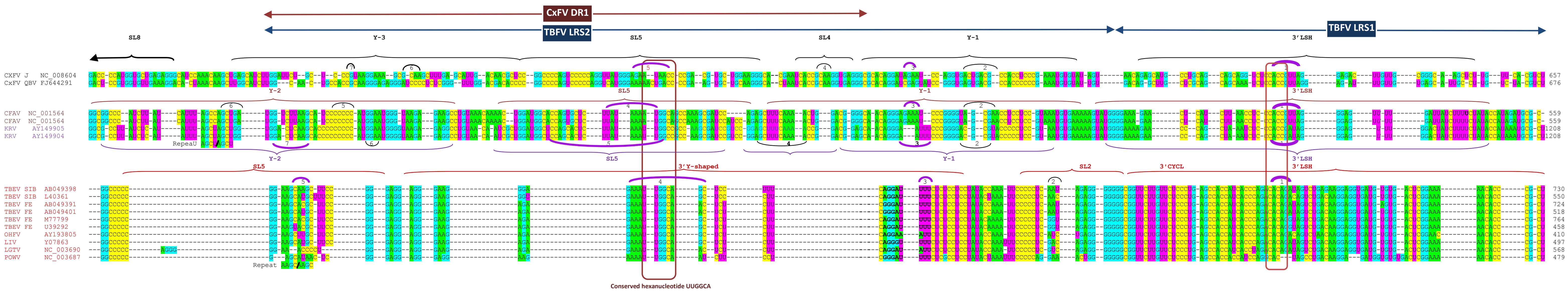

Figure S4C. Alignment between TBFV LRS3-4 and ISFV DRs1-4/R1-2

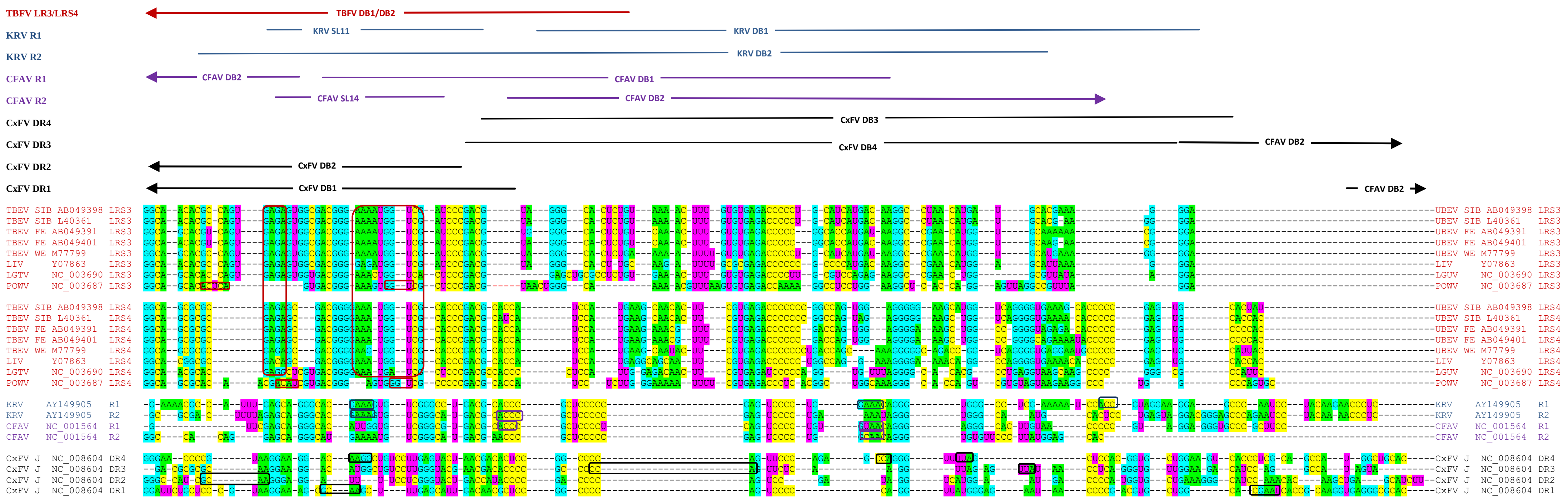

Supplement: Figure S4 — ISFV 3′UTR alignments (to view with magnification 130–200%). Viruses are identified by the abbreviated names and accession numbers. The R1 and R2 correspond to 67-nt long DRs of the KRV and CFAV identified in [7]. A. The alignment between 3′UTR of Aedes- (KRV and CFAV, black letters) and of Culex-associated (red letters) ISFV annotated with RNA conformations (oval brackets) displayed in Figure S3. The boundaries of extended CxFV DR1-DR4 are indicated in blue arrowed lines. B. Alignment between TBFV and ISFV annotated with secondary RNA structures as scheduled in Figures S1 and S3 in the region of the CxFV 5′DR1-3′LSH. Red semi-oval brackets specify loops with sequences similar between TBFV and ISFV. The regions of CxFV DR1 and TBFV LRS2-LRS3 are indicated. C. Alignment between TBFV LRS3–4 and ISFV DRs1–4/R1–2. The loop sequences of TBFV DB1 and DB2 are enclosed in ovals. Solid lines, with appropriate colour code, outline the positions of the DB-like and SLs encoded by the repeated regions; the arrows indicate the extension of the DB-like structure outside the alignment region shown. (PDF) [file pone.0092056.s004.pdf]
