# Supplementary material for: Molecular Archaeology of Flaviviridae Untranslated Regions: Duplicated RNA Structures in the Replication Enhancer of Flaviviruses and Pestiviruses Emerged via Convergent Evolution
Source: PLoS One. 2014 Mar 19;9(3):e92056. doi: 10.1371/journal.pone.0092056 (PMC3960163; doi:10.1371/journal.pone.0092056)

Figure S5. Convergent duplications of flavivirus and pestivirus sequences in the 3'UTR.

Figure S5A

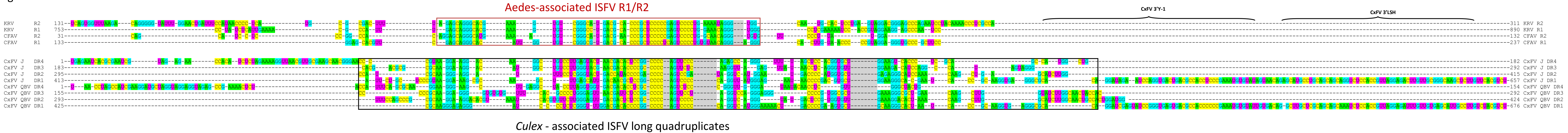

Figure S5B

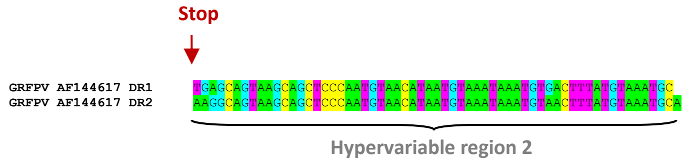

Supplement: Figure S5 — Convergent duplications of flavivirus and pestivirus sequences in the 3′UTR. A. Independent origin of Aedes (KRV/CFAV) R1/R2 (red-box) and Culex DRI-DR4 (black-box) duplicated ISFV sequences. Virus 3′UTR sequences are enumerated from the stop codon of the single ORF. The CxFV 3′LSH and 3′Y-1 structures are mapped to the alignment. The Culex- and Aedes-specific gaps are enclosed in grey areas. The full-length of the Culex- associated ISFV 3′UTR is presented whilst only aligned fragments of KRV and CFAV 3′UTR are shown. B. Sequence identity of GRFPV duplications. (PDF) [file pone.0092056.s005.pdf]
