# Supplementary material for: Molecular Archaeology of Flaviviridae Untranslated Regions: Duplicated RNA Structures in the Replication Enhancer of Flaviviruses and Pestiviruses Emerged via Convergent Evolution
Source: PLoS One. 2014 Mar 19;9(3):e92056. doi: 10.1371/journal.pone.0092056 (PMC3960163; doi:10.1371/journal.pone.0092056)
Supplement: Figure S7 — Predicted secondary RNA structures for the pestiviruses. Images were produced by MFold using MDBP = 60 for A) HoCV; B) CSFV; C) RNDPV; D) GRFPV; E) BDV; F) BDVD2; G)BVDV1. Viruses are identified by the abbreviated names and accession numbers. The SLs are numerated and annotated with the conserved 3′CPN (highlighted in red), conserved loop 2 sequences (highlighted in bright green) and conserved ssRNA region (blue line). The DRs of each pestivirus are highlighted in identical colours. (PDF) [file pone.0092056.s007.pdf]

Figure S7A
HoCV
AF091507

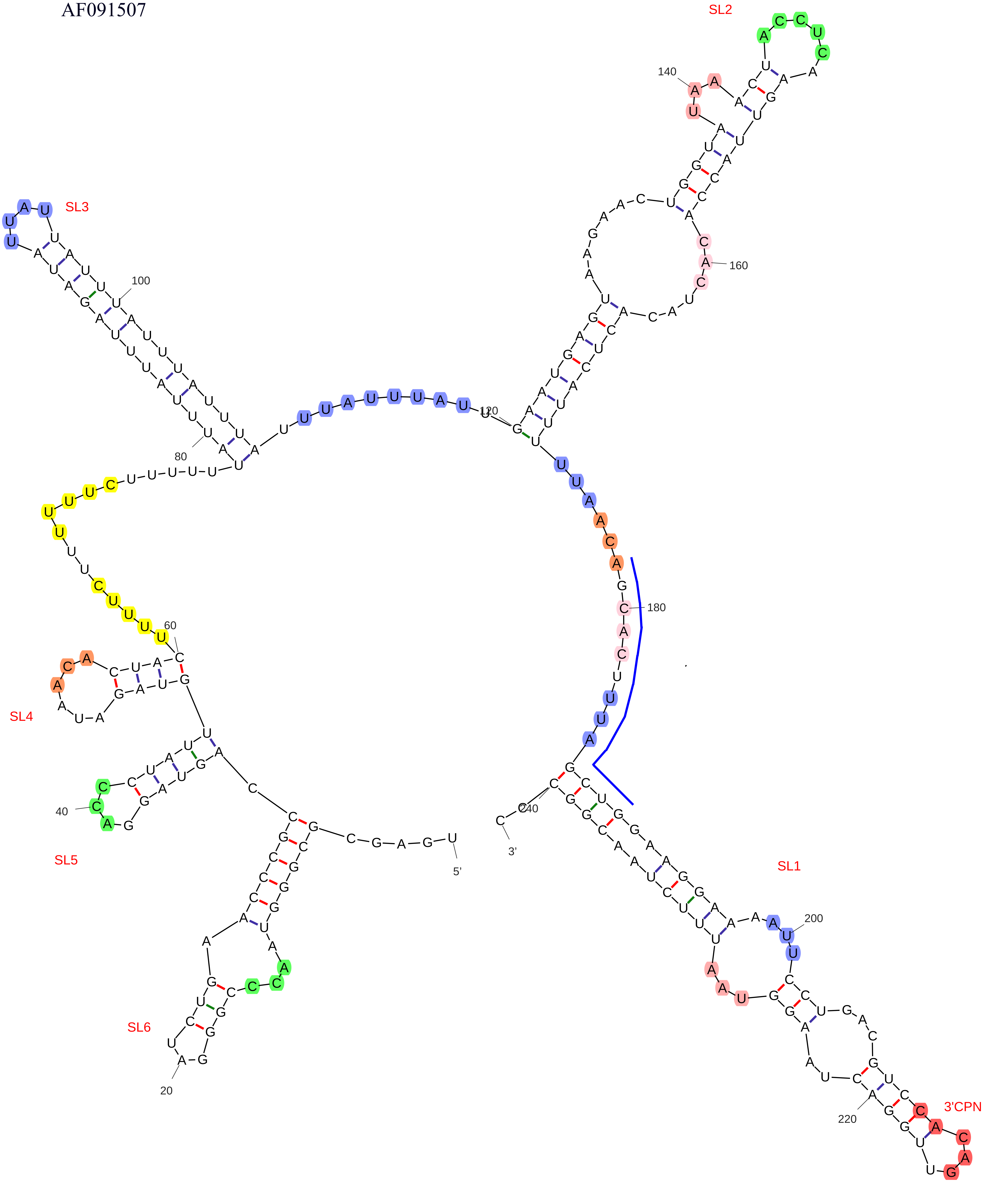

dG = -49.40 [Initially -47.90]

Figure S7B
CSFV
EU503187

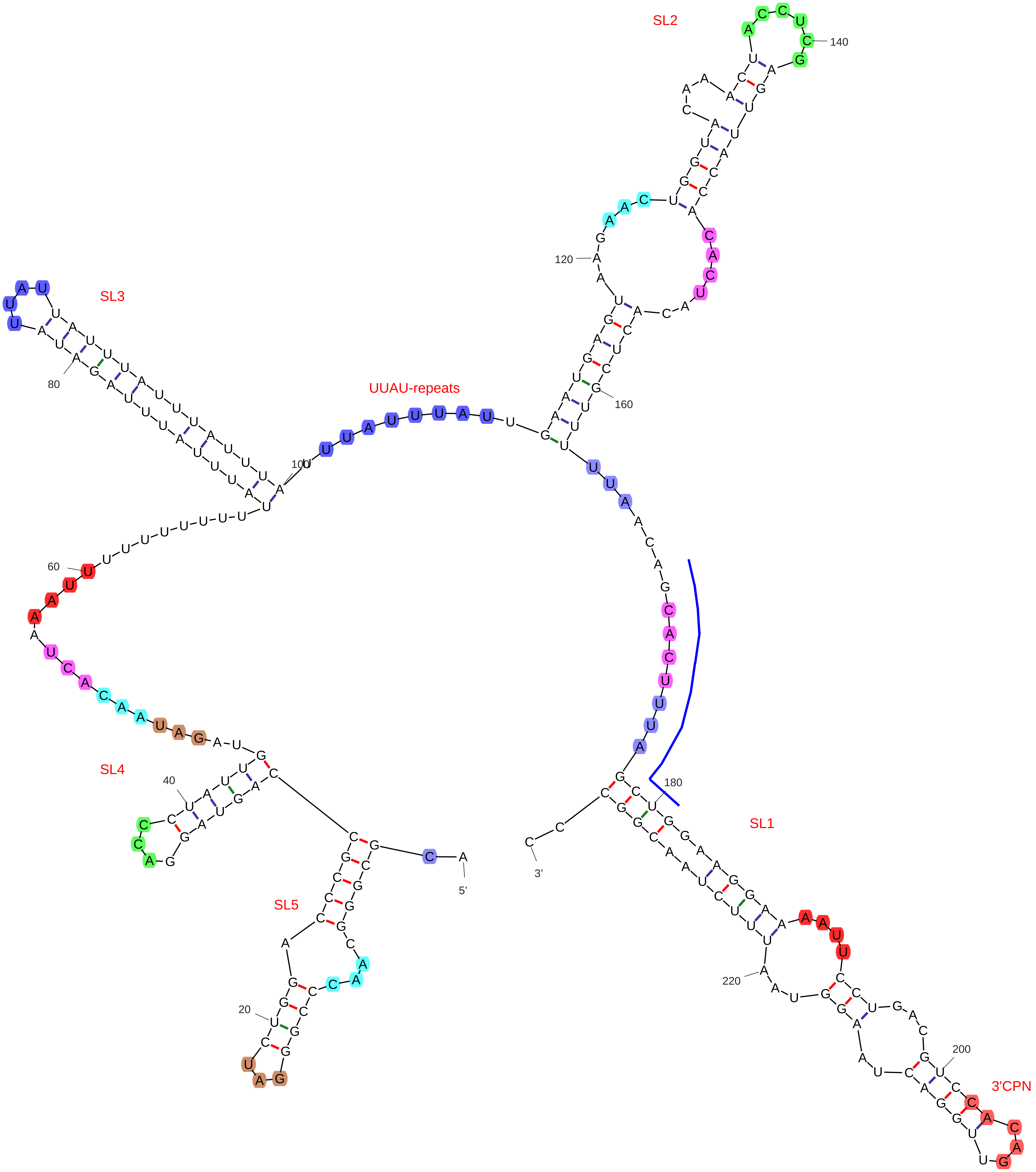

dG = -51.70 [Initially -49.30]

Figure S7C
RNDPV
AF144618

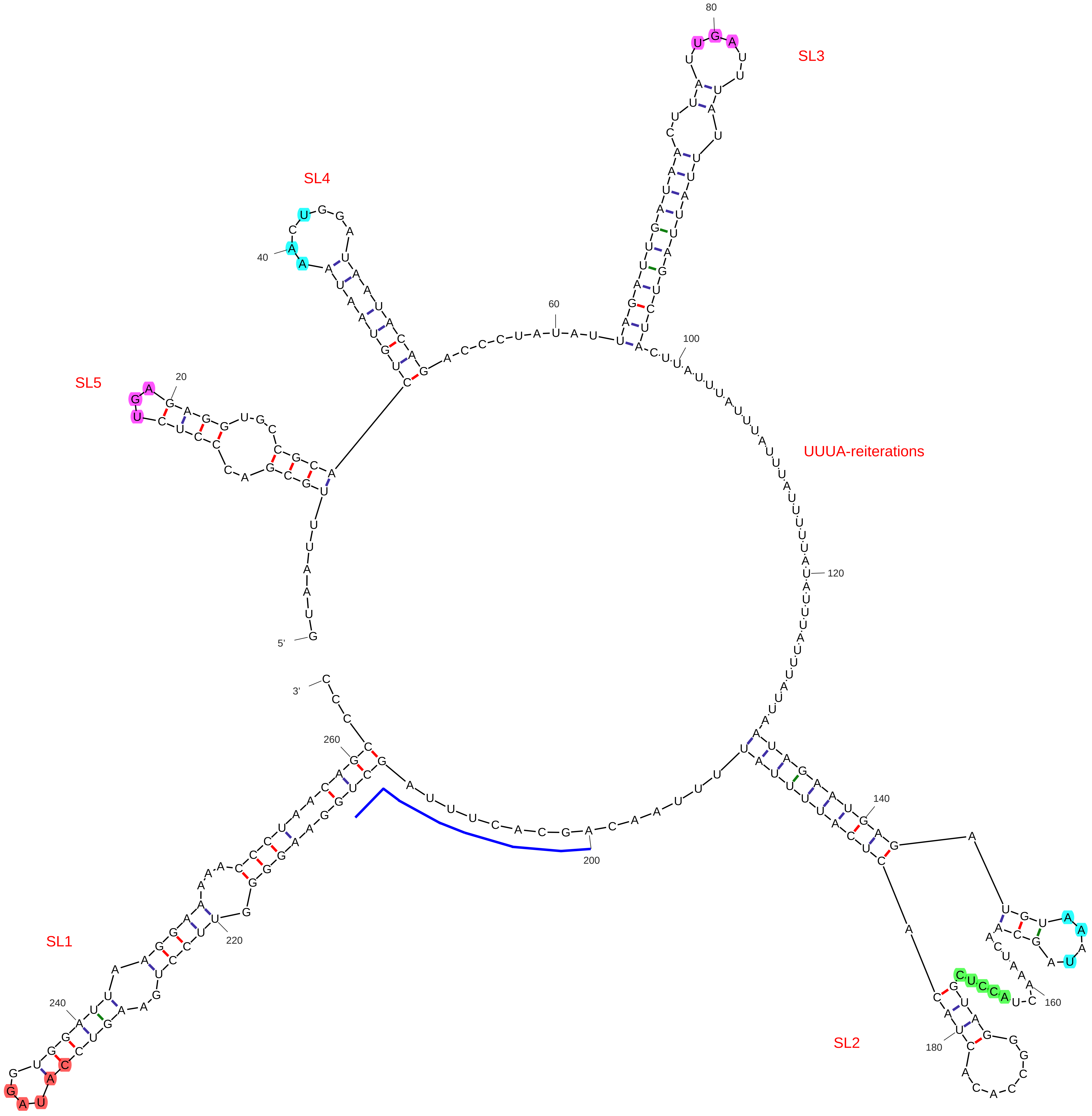

dG = -53.29 [Initially -56.00] I

Figure S7D
GRFPV
AF144617

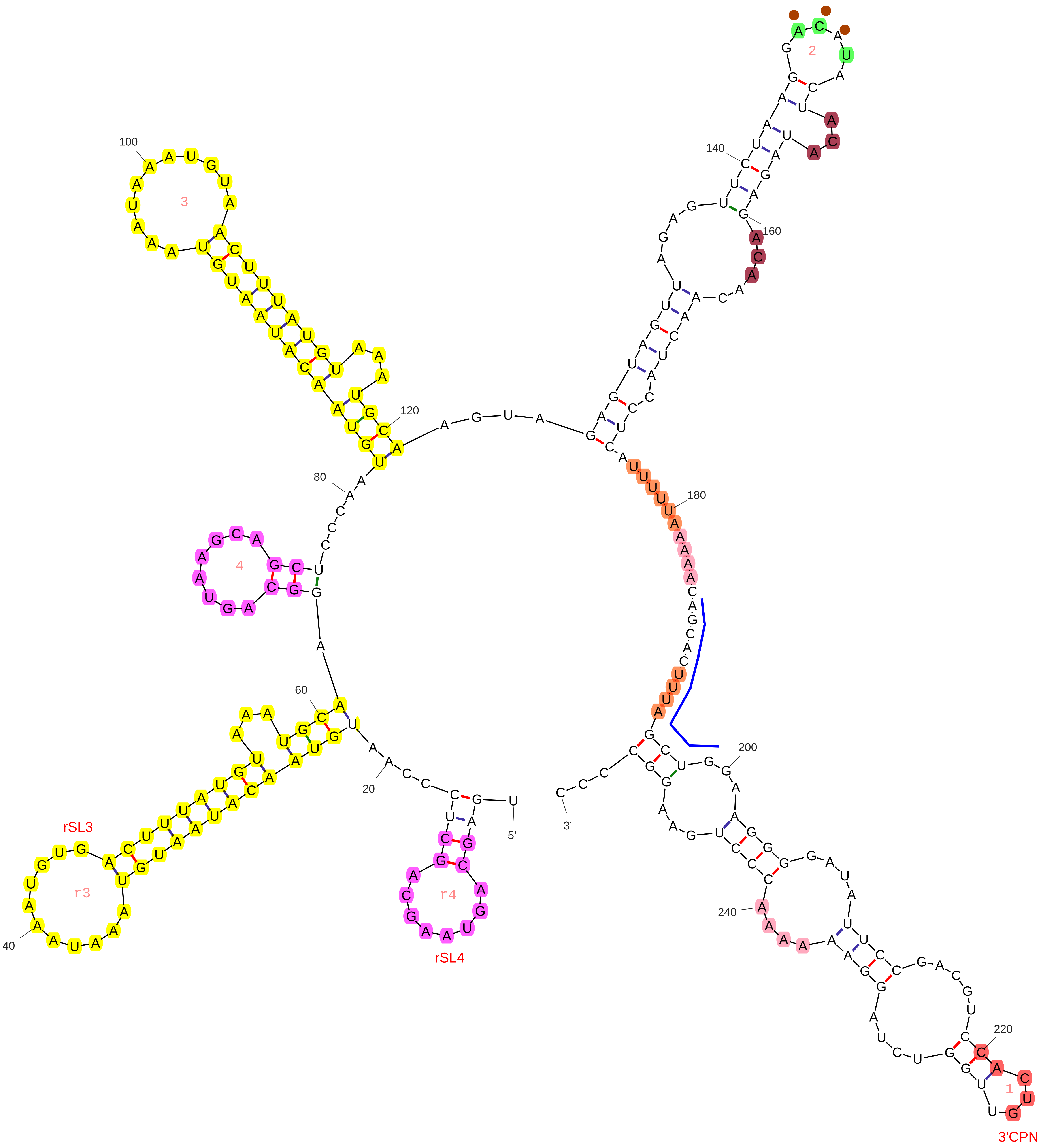

dG = -44.20 [Initially -41.90]

Figure S7E
BDV
NC_003679

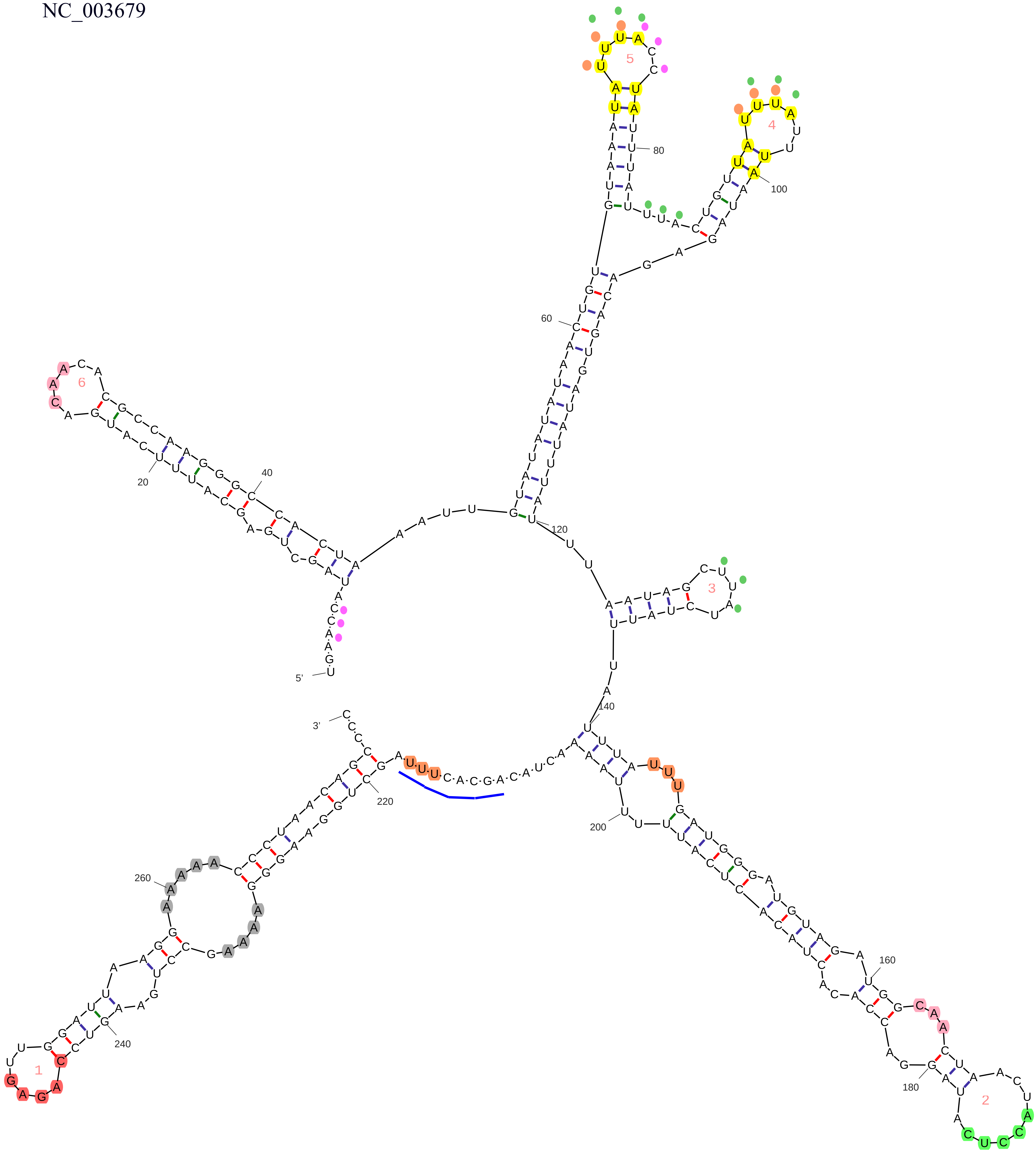

dG = -49.00 [Initially -51.60]

Figure S7F
BVDV2
AY149215

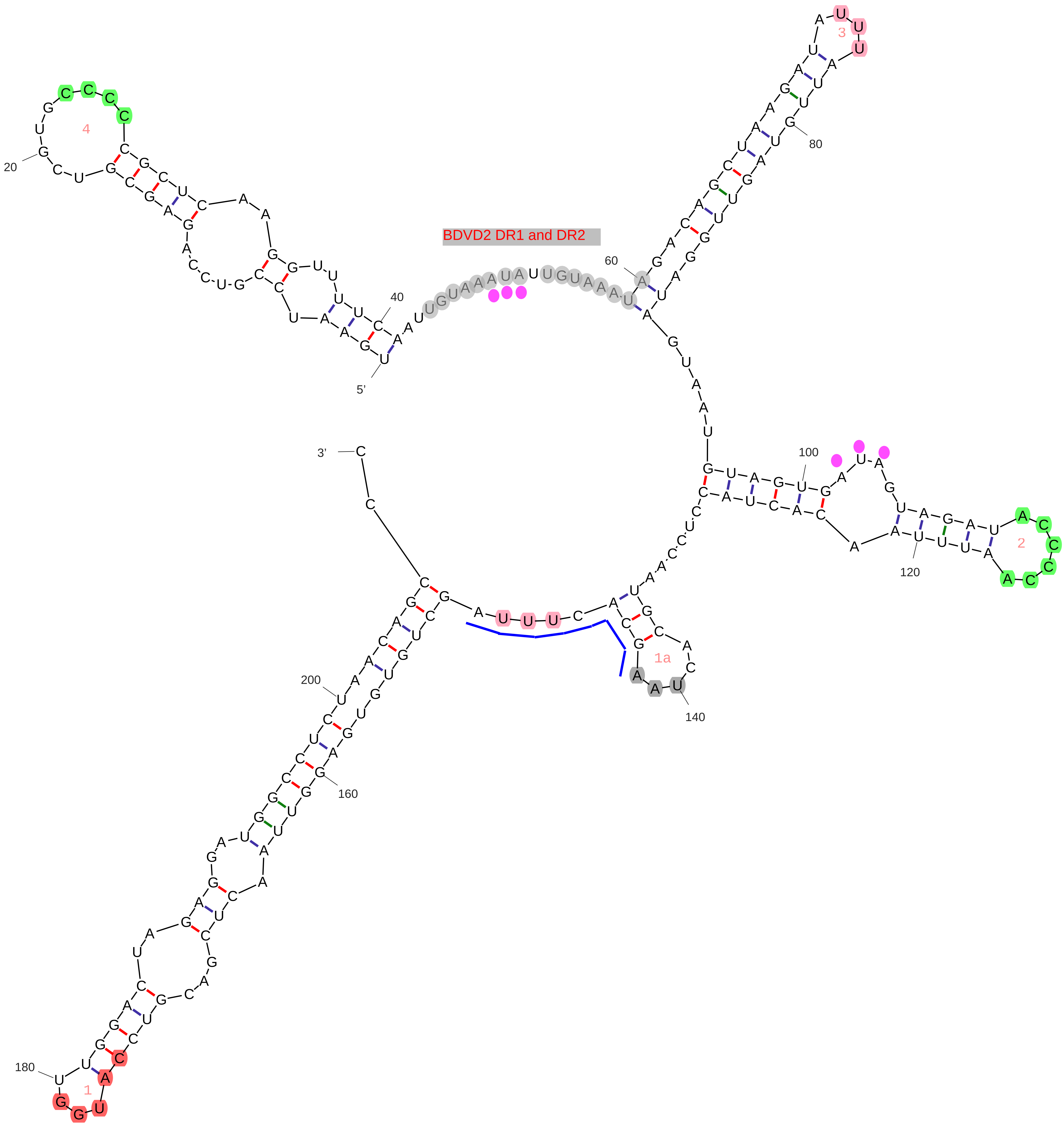

dG = -52.00 [Initially -52.00]

Figure S7G
BVDV1
AF037411

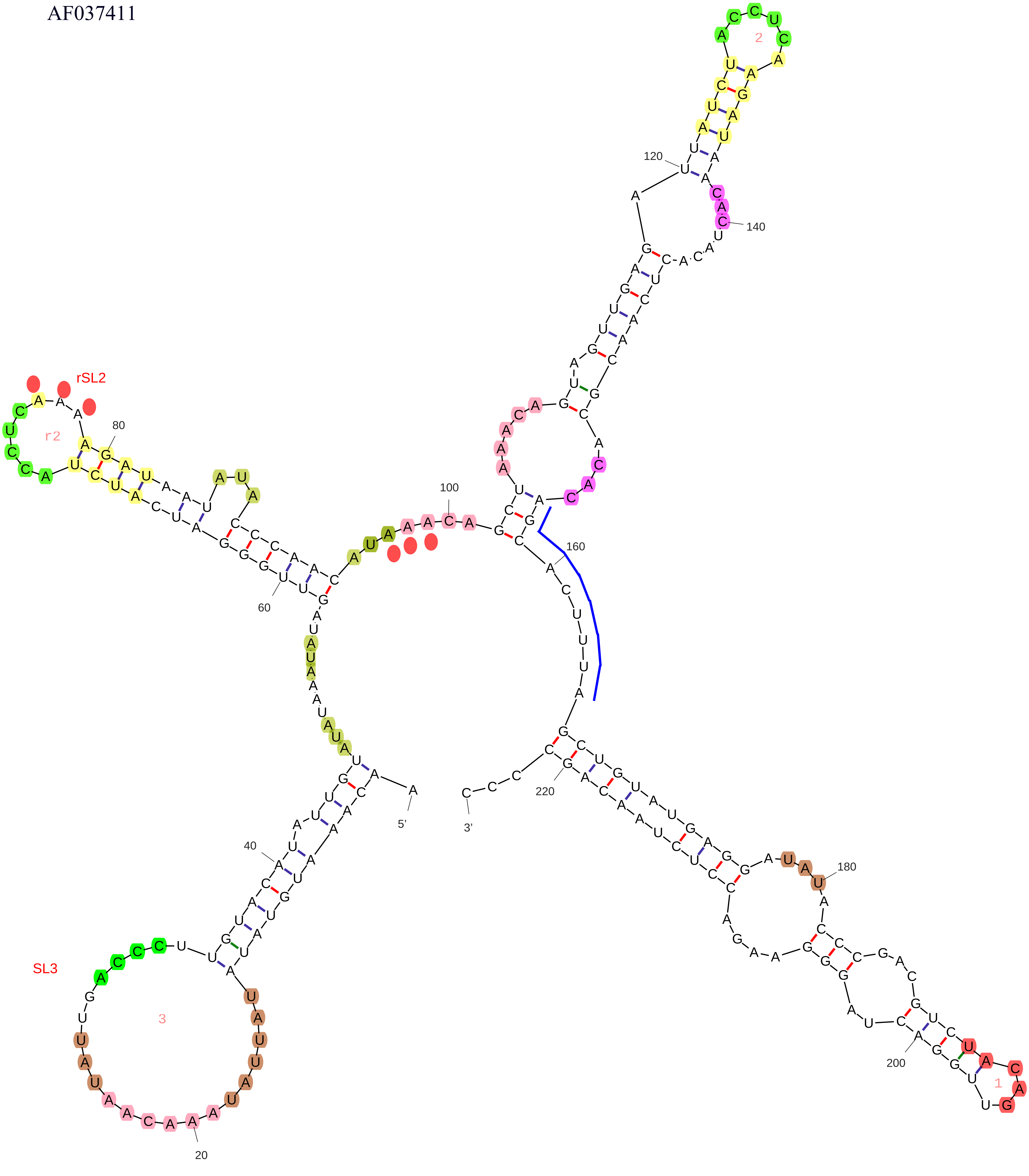

dG = -47.30 [Initially -47.30]
