# Supplementary material for: Molecular Archaeology of Flaviviridae Untranslated Regions: Duplicated RNA Structures in the Replication Enhancer of Flaviviruses and Pestiviruses Emerged via Convergent Evolution
Source: PLoS One. 2014 Mar 19;9(3):e92056. doi: 10.1371/journal.pone.0092056 (PMC3960163; doi:10.1371/journal.pone.0092056)
Supplement: Table S1 — List of abbreviations. (PDF) [file pone.0092056.s008.pdf]

**Supporting Table 1. List of abbreviations.**

| | |
|---|---|
| 3’CPN | Conserved pentanucleotide CACAG |
| 3’CR | 3’ Conserved region |
| 3’HVR | 3’ Hypervariable region |
| 3’UTR | 3’untranslated region |
| 3’VR | 3’ Variable region |
| 3'LSH | Long stable hairpin |
| 5’UTR | 5’untranslated region |
| AHFV | *Alkhumra haemorrhagic fever* virus |
| BDV | *Border disease virus* |
| BVDV | *Bovine diarrhoea virus* |
| C3'UTR | Conserved 3'UTR region |
| CFAV | *Cell Fusion Agent virus* |
| CSV | *Classical swine fever virus* |
| CxFV | *Culex flavivirus* |
| DB | Dumbbell-like structure |
| DENV | *Dengue virus* |
| DRs | Direct repeats |
| GRFPV | *Giraffe pestivirus* |
| HoCV | *Hog Cholera virus* |
| ISFV | Insect-specific flaviviruses |
| ISG | Interferon-stimulated gene |
| JEV | *Japanese encephalitis virus* |
| KFDV | *Kyasanur Forest disease virus* |
| KRV | *Kamiti River virus* |
| LGTV | *Langat* |
| LIV | *Louping ill virus* |
| LRS | Long repeated sequences |
| MBFV | Mosquito-borne flaviviruses |
| MDBPB | Maximal distance between paired bases |
| NFAR | Nuclear factor associated proteins |
| NKV | No-known vector |
| $NS5^{pol}$ | NS5 polymerase |
| OHFV | *Omsk haemorrhagic fever virus* |
| pLRS | Primordial LRS |
| POWV | *Powassan virus* |
| PVs | Pestiviruses |
| R | Repeat |
| RdRp | RNA-dependent RNA polymerase |
| RNDPV | *Reindeer pestivirus* |
| sfRNA | Subgenomic flavivirus RNA |
| SLs | Stem-loops |
| TBEV | *Tick-borne encephalitis virus* |
| TBFV | Tick-borne flaviviruses |
| V3'UTR | Variable 3'UTR region |
| WNV | *West Nile virus* |
| YFV | *Yellow fever virus* |
